# On the use of the serial dilution culture method to enumerate viable phytoplankton in natural communities of plankton subjected to ballast water treatment

**DOI:** 10.1007/s10811-015-0601-x

**Published:** 2015-05-24

**Authors:** John J. Cullen, Hugh L. MacIntyre

**Affiliations:** Department of Oceanography, Dalhousie University, Halifax, Nova Scotia Canada B3H 4R2

**Keywords:** Most probable number, MPN, Extinction dilution method, Viability, Vitality, Enumeration, Invasive species, Ultraviolet radiation, Vital stains

## Abstract

Discharge standards for ballast water treatment (BWT) systems are based on concentrations of living cells, for example, as determined with vital stains. Ultraviolet radiation (UV) stops the reproduction of microorganisms without killing them outright; they are living, but not viable, and ecologically as good as dead. Consequently, UV-treated discharge can be compliant with the intent of regulation while failing a live/dead test. An alternative evaluation of BWT can be proposed based on the assessment of viable, rather than living, cells in discharge water. In principle, the serial dilution culture-most probable number (SDC-MPN) method provides the appropriate measure for phytoplankton. But, the method has been criticized, particularly because it is thought that many phytoplankton species cannot be cultured. A review of the literature shows that although SDC-MPN has been used for more than 50 years—generally to identify and count phytoplankton species that cannot be preserved—its application to enumerate total viable phytoplankton seems to be new, putting past criticisms of the method in a different light. Importantly, viable cells need to grow only enough to be detected, not to be brought into sustained culture, and competition between species in a dilution tube is irrelevant as long as the winner is detectable. Thorough consideration of sources of error leads to recommendations for minimizing and quantifying uncertainties by optimizing growth conditions and conducting systematic comparisons. We conclude that with careful evaluation, SDC-MPN is potentially an effective method for assessing the viability of phytoplankton after BWT.

## Introduction

In response to the threats from continued introductions of aquatic invasive species, the United Nations International Maritime Organization (IMO) adopted the *International Convention for the Control and Management of Ships’ Ballast Water and Sediments* (IMO 2004). The convention has yet to be ratified, but the US Coast Guard (USCG) has established national regulation of systems to “kill, render harmless, or remove” organisms from ballast water discharge (US Coast Guard 2012). Ships will have to treat ballast water to meet regulatory discharge standards. Two size classes of plankton are subject to regulation, classified by size: ≥50, and ≥10 and <50 μm (IMO 2004; US Coast Guard 2012). To meet the standard, any ballast water management system (BWMS) must discharge <10 “living” cells mL^−1^ (US Coast Guard 2012) or <10 “viable” cells mL^−1^ (IMO 2004) in the 10–50 μm size range and <10 “living” or “viable” cells m^−3^ in the >50 μm size range. The USCG acknowledges that the two standards are slightly different (US Coast Guard 2012) but points out that for the purpose of their approval guidelines, the IMO defines “viable” as “living” (see International Maritime Organization Marine Environment Protection Committee 2008).

The distinction between viable, which for our discussion we define as being reproductive, and living, i.e., showing signs of vitality, has important implications for the evaluation of ballast water treatment (BWT) systems. One method, irradiation with ultraviolet radiation (UV, particularly ultraviolet-C), is a proven and widely-applied technology for disinfection of wastewater and drinking water (Hijnen et al. 2006) that inactivates microbes by destroying their ability to reproduce but without necessarily killing them outright. As a result, cells that have been effectively treated with UV can be intact and metabolically active—that is, living—but incapable of reproduction and thus nonviable (First and Drake 2013a). Consequently, organisms that have been rendered harmless through treatment with UV would be compliant with the intentions of BWT regulations but living and thus noncompliant according to the regulations themselves.

Since a living, but nonreproductive, microbe is ecologically as good as dead (i.e., it is not a viable propagule, a term used by Reavie et al. 2010), it can be argued that viability is inherently more accurate than vitality as a measure of invasive potential and that alternative BWT regulations based on viable cells, as compared with living cells, should provide equal protection to the environment while allowing the effectiveness of UV treatment systems at rendering cells harmless to be assessed more accurately. This argument is relevant because the Environmental Technology Verification (ETV) protocol includes consideration and utilization of alternative methods after they have been validated (ETV 2010). But, an important question must be considered: Can the concentration of viable cells in natural plankton communities be measured reliably? Addressing the issue in 2012, the USCG opted to use live/dead rather than viable/nonviable as a regulatory criterion, because the determination of viability would require culturing potentially large numbers of different kinds of organisms, many of which, they claimed, scientists are not able to culture (US Coast Guard 2012). Consistent with the reasoning that all dead cells are also nonviable and thus noninvasive, the Coast Guard further supported their decision by pointing out that live/dead is more conservative, and thus more protective, than viable/nonviable. But, because the UV doses required to kill microbes greatly exceed those required to inactivate them—past the point of economic practicality--the live/dead criterion could effectively exclude UV technology from being used to treat ballast water.

A recent study by First and Drake (2013a) frames the live/dead/viable issue in the context of assessing the effectiveness of UV for BWT. Arguing that viability is the appropriate measure of invasive potential, they assert that direct measures of growth after treatment (“regrowth assays,” e.g., Liebich et al. 2012) are definitive. But, they point out that such assays are time-consuming, lasting days to weeks, and that the method applies only to organisms that will grow under laboratory conditions, but that many microorganisms cannot be cultured. The regrowth method employed by Liebich et al. (2012) and others with similar aims (Wright et al. 2009) tracked the growth of cells after BWT, but they did not estimate the initial concentration of total viable cells per mL immediately after treatment, the measure most relevant to discharge regulations.

Many assays classify single cells according to signs of life (Zetsche and Meysman 2012; Steinberg et al. 2012; Reavie et al. 2010), including vital stains (Steinberg et al. 2011; Reavie et al. 2010; Zetsche and Meysman 2012), a mortal stain (Steinberg et al. 2012; Reavie et al. 2010), cellular integrity (Burkholder et al. 2007; Wright et al. 2009), motility (Gregg and Hallegraeff 2007), and indicators of cell division (discussed by First and Drake 2013a). Each of these measures is unquestionably related to vitality or viability, but none have been related systematically (i.e., across taxa and subject to varying degrees of debilitation) to quantitative measures of the capability of microbes to reproduce after BWT.

Bulk measurements, such as metrics of variable chlorophyll fluorescence (Drake et al. 2014) and changes in chlorophyll concentration (e.g., Wright et al. 2009), have been examined as measures of the effects of BWT on phytoplankton. Zetsche and Meysman (2012) argue that the existing regulations based on cell counts preclude the use of such bulk measurements for testing, but Drake et al. (2014) present a validation framework for compliance monitoring that would use bulk measurements when they are related quantitatively to concentrations of cells that satisfy the regulatory criterion—for their examples, living cells. For proxies of the ability to reproduce, such bulk measurements would have to be related to concentrations of viable cells. Even if they do not provide direct estimates of living or viable cells in discharge water, rapid assays based on bulk measurements or single-cell indicators can be important in shipboard testing for compliance with discharge regulations (King and Tamburri 2010).

**Fig. 1:**
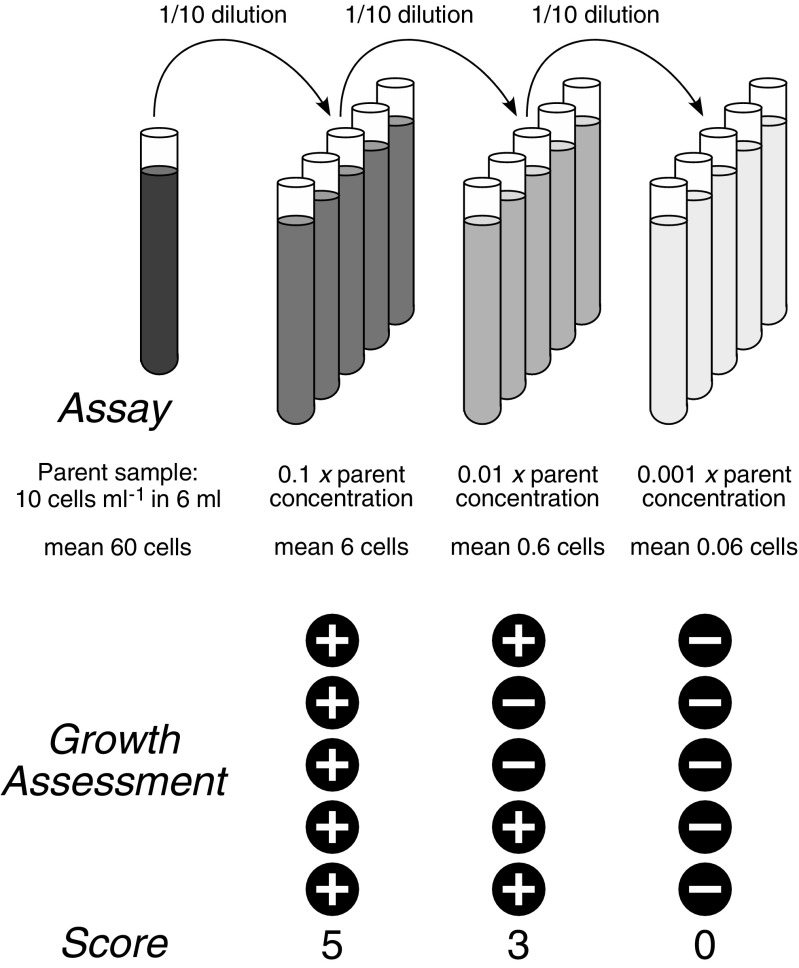
Principles and assumptions of the serial dilution culture-most probable number (SDC-MPN) method. The SDC-MPN method (Throndsen 1978) estimates the concentration of viable cells in a sample, based on incremental dilution of the sample into a series of replicated liquid subcultures (e.g., test tubes) and statistical determination of the hypothetical dilution that corresponds to one viable cell per subculture tube. Back calculation gives the number of viable cells in the parent sample tube; the concentration of viable cells (cells mL^−1^) is determined from that number and the volume of culture in the tube. The test is based on the discrimination of subcultures containing one or more viable cells (assumed for now to result in a positive score for growth) from those with none (a negative score). The proportion of tubes scoring positive at any given dilution is a function of the concentration of viable cells in the parent culture and the dilution factor. It is assumed (Cochran 1950; Haas and Heller 1988) that (i) organisms are randomly distributed in each tube and evenly distributed between subsamples and (ii) growth will be reliably detected in any tube containing one or more viable phytoplankton cells. • In principle, all replicated tubes expected to have ≫1 viable cell (low dilution) would show growth and score positive. At higher dilutions, negative scores become more likely until at very high dilutions (calculated concentration < < 1 per tube), the number of positive scores declines to zero. • Among *n* replicate tubes at any dilution, the likelihood of *s* negative scores follows the binomial distribution set by the probability of a tube being sterile; the most likely number of positive scores thus corresponds to the probability of a tube having one or more viable cells (Cochran 1950). • In the illustrated example of a parent tube of 6 mL containing 60 cells (10 cells mL^−1^), the calculated number of viable cells per tube in each successive tenfold dilution is 6, 0.6, and 0.06 respectively. In a five-replicate tube test, the most likely number of positives at each respective dilution is therefore expected to be 5, 3, and 0, but other combinations of scores are possible due to random chance. • The calculation of MPN, with 95 % confidence intervals (CI), is based on statistical comparison of observed scores with calculated probabilities. The MPN corresponding to the test score can be calculated (e.g., Hurley and Roscoe 1983; Garthright and Blodgett 2003) or found in lookup tables (Blodgett 2010); dilutions need not be constrained to tenfold intervals. In the illustrated example for a score of 5, 3, 0, the MPN is 79 cells and expressed as a concentration is 13 cells mL^−1^ (=79 cells/6 mL). The 95 % CI, based on the logarithm of the estimate, is 4–37 cells mL^−1^. The confidence intervals decrease if the number of replicate tubes at each dilution is increased or if more dilutions, especially with smaller dilution factors (e.g., 5×) are used; larger tube volumes increase the sensitivity of the assay

Clearly, there is a need for a method to enumerate viable cells in a sample of plankton. As we will discuss in more detail below, the most direct method for phytoplankton is the serial dilution culture-most probable number assay (SDC-MPN, also called the extinction dilution method, Throndsen 1978) (Fig. 1). The approach is based on a bacteriological assay developed more than a century ago (McCrady 1915; Cochran 1950), which was applied to phytoplankton in 1951 (Knight-Jones 1951). The SDC-MPN method is considered to be problematic when applied to natural communities of phytoplankton, however, in large part because many planktonic microorganisms are assumed to be unculturable (Steinberg et al. 2011; First and Drake 2013a; US Coast Guard 2012) and also because of concerns about interactions between species^1^ in the dilution cultures. Reflecting such concerns, the US Environmental Protection Agency ETV Program (2010) generic protocol for the verification of BWT technology cautioned that the MPN method that they used on cultures of phytoplankton (see also Oemcke and Van Leeuwen 2005) “is suitable for pure cultures of heterotrophic protists or phytoplankton, but is not a useful tool for mixed cultures.” Here, we revisit prior assessments of the method.

## Framework of the review

### Motivation

Sharing the widely held misgivings about SDC-MPN for natural communities but considering the demonstrated need for a robust assay of the concentration of viable phytoplankton in treated ballast water, we have reviewed the principles, assumptions, and applications of SDC-MPN. Our investigation has led us to a new appreciation of the method and its application in BWT. The ETV protocol (2010) allows the consideration of alternative methods for measuring the concentration of living organisms in discharge water. Based on our review, we suggest that SDC-MPN may serve as an alternative to the existing method based on vital stains.

### Fundamental postulate

Significantly, the SDC-MPN method enumerates viable phytoplankton but discharge regulations as they stand specify concentrations of living cells. This important distinction would have to be accommodated if the SDC-MPN method were to be adopted for use in regulations. This is not our decision to make. Rather, we present at the outset a fundamental postulate, based on the intent of BWMS regulations:

Because neither a dead organism nor a nonreproductive organism can propagate after discharge from ships’ ballast, discharge criteria based on vitality (live/dead) and viability (the ability to reproduce) are equally protective of coastal environments.

This will be referred to as the postulate of equivalent protection.

### Approach

Guided by this postulate, we classify potential errors in ballast water testing according to their implications for protection of the environment, not how they conform to live/dead regulatory criteria. In this context, we review the SDC-MPN method for phytoplankton and identify sources of inaccurate results; we discuss their likely influence on the estimation of total viable phytoplankton cells in natural communities of plankton before and after ballast water treatment, and how the resulting errors can be estimated and reduced.

### Scope

Given its demands for time, SDC-MPN would be appropriate for land-based verification to gain type approval for ballast water management systems, not rapid assays that are required for shipboard compliance testing (King and Tamburri 2010). The method enumerates only photoautotrophs, and its utility is primarily for counting viable cells in the 10–50 μm size range that are not readily removed by filtration during BWT. We discuss briefly approaches that can be used to assess other components of the plankton, such as heterotrophs, and the use of SDC-MPN on cultures of phytoplankton to develop proxies of viability for rapid assays that would be suitable for shipboard compliance testing and port state control inspection.

### Objective

We intend to support an argument that the SDC-MPN method can potentially produce ecologically valid assessments of the effectiveness of ballast water treatment that are constrained with reasonable estimates of uncertainty, especially if sources of error are specifically assessed.

## Background

Well over 100 years ago, the dilution method was established as one of several techniques for isolating phytoplankton for growth in unialgal culture (Allen and Nelson 1910). The authors dispensed one or two drops of a plankton sample into petri dishes containing, for example, 250 mL of growth medium. After a few days, colonies of diatoms appeared, likely originating from individual cells in the dilute culture; they could easily be isolated by pipette and inoculated into fresh medium, ultimately to be maintained in unialgal culture.

Convinced that centrifugation—an established method for concentrating small phytoplankton in natural samples for microscopic enumeration—greatly underestimated the numbers of small cells that could not be retained by meshes, and recognizing that species that grew in diluted cultures represented at least one cell in the inoculum, Allen (1919) estimated the minimum numbers of cells in samples from the Knap Buoy station near Plymouth using a dilution method. He diluted 0.5 mL of sea water in 1.5 L of growth medium and divided the mixture between 70 flasks, each containing about 21 mL, that were “placed in a north light and kept at room temperature without a fire” and examined periodically over the following 6 weeks. Altogether, he found 232 different organisms distributed among the 70 flasks. He concluded that this was the minimum number of cells in the original 0.5 mL, so the concentration of phytoplankton was at least 464 cells mL^−1^. Allen emphasized that the real count would be considerably higher than this because more than one cell of the same species might have been introduced into some flasks and also because not all species could grow under the conditions he provided. Even so, the count was more than 30 times that from a centrifuged sample. Allen concluded that no one approach could provide quantitative estimates of phytoplankton concentrations, so a variety of methods would have to be used to obtain accurate counts.

To support a study on the systematics and abundance of ultraplankton and nanoplankton, many of which are difficult to identify in a counting chamber and do not preserve well for enumeration, Knight-Jones (1951) used a dilution culture method to isolate and enumerate natural phytoplankton. The enumerations were based on procedures used by the UK Ministry of Health for bacteriological assays of water supplies, using statistical tables to estimate counts and errors. Three serial dilutions were prepared with Erd-Schreiber medium, each with five tubes, and these were incubated in windows facing north for 1 to 4 months as he waited for cultures to develop. Knight-Jones (1951) tabulated probable numbers for total phytoplankton and also used the same tables to estimate the concentrations of individual species. He found that a 1.5-μm flagellate, now classified as the prasinophyte *Micromonas pusilla*, was the most generally abundant—remarkable because the organism had been previously undescribed. The importance of suitable culture conditions, difficult to provide at the time, was highlighted. Knight-Jones concluded, “If a thermostatically-controlled culture-cabinet were used, quantitative culturing would appear to be a very practicable method of nanoplankton estimation.” (p. 154).

The SDC-MPN method was subsequently refined and described in the *Intergovernmental Oceanographic Commission–UNESCO Phytoplankton Manual* by Throndsen (1978) and updated by Andersen and Throndsen (2003), who highlighted that the method is useful for the isolation of phytoplankton while offering “the opportunity to make estimates of the original cell number.” Indeed, over the years, SDC-MPN has been applied in studies that required isolation of phytoplankton cultures as well as enumeration of the starting concentrations of taxa from which they came. Modifications of the method have also been applied to enumerate propagules of phytoplankton in sediments (Ishikawa and Furuya 2004), to estimate the concentrations of protozoa in sea water (Lighthart 1969) and to isolate and enumerate phages (Suttle and Chan 1993).

Serial dilution culture has been particularly useful in the enumeration of groups of phytoplankton that do not preserve well, such as flagellates and monads in the subsurface chlorophyll maximum (Furuya and Marumo 1983), naked nanoflagellates in the Kiel Bight and Kiel Fjord (Jochem 1990), and *Micromonas pusilla* as part of the nanoplankton communities of the Bering Sea (Throndsen and Kristiansen 1991).

Throndsen and Kristiansen (1991; see also Backe-Hansen and Throndsen 2002) identified sources of error in the SDC-MPN method when they applied it to identify and enumerate co-occurring species in samples: lack of growth of some specimens introduced into the tubes, competitive relations in a single tube, the influence of culture conditions and the medium on growth, and insensitivity of the method for detecting heterotrophs. We return to these issues in following sections.

A study of the diversity and seasonality of cryptomonads in the Gulf of Naples (Cerino and Zingone 2006) illustrates strengths and weaknesses of the SDC-MPN method when it is used to describe community composition of the phytoplankton. Although they are identifiable by diagnostic pigments in bulk samples (Gieskes and Kraay 1983) and fluorescence and cell-size signatures detectable with flow cytometry (Li and Dickie 2001), the diversity of marine cryptomonads is scarcely known because generally they are not reliably identifiable to species under light microscopy and they are usually damaged by common fixatives (Kugrens and Lee 1987). But, by growing cryptomonads in culture using SDC-MPN, Cerino and Zingone (2006) were able to identify reliably and describe patterns in abundances of cryptomonad species, in the process bringing about 80 strains into culture for more thorough examination. The authors acknowledged that the SDC-MPN method is subject to error when competition eliminates culturable specimens in some tubes; still, their method yielded information on diversity that could only be obtained by growing and identifying isolates. Flow cytometry with cell sorting offers alternatives to serial dilution, both for isolation of cultures (Sieracki et al. 2005; Sinigalliano et al. 2009) and genomic quantification of biodiversity without the need for culturing (Kashtan et al. 2014; Heywood et al. 2010), but it has yet to be examined as a method for quantifying total viable cells in natural samples of phytoplankton.

Inspection of type approvals for BWT systems (IMO 2015) reveals that SDC-MPN has been used in regulatory testing for the 10–50 μm size class of phytoplankton, but it is difficult to review the method specifically for the assessment of ballast water treatment because much of the information is outside the standard scientific literature (Albert et al. 2013). When the serial dilution culture method is mentioned in publications about BWT, it is usually to point out its limitations, described above. However, First and Drake (2013b) conducted SDC-MPN experiments on natural phytoplankton in the ≥10 and <50 μm size class, concentrated by screening. The method was used to enumerate viable phytoplankton before and after samples were treated with two doses of UV (200 and 500 mJ cm^−2^) in each of three experiments. The MPN estimates of viable phytoplankton cells in the untreated controls were not significantly different from initial cell counts, consistent with most of the cells being viable and accurately detected as such, but measured survival was low in the treated samples: Viable phytoplankton were detected after UV treatment in only one of the three experiments, and only at the lower of the two doses. Detailed discussion of the SDC-MPN method or its results was not warranted in this study, which focused on other approaches for ballast water testing (First and Drake 2013b). Others have followed the growth of phytoplankton species in natural samples after treatment (Wright et al. 2009; Liebich et al. 2012), and SDC-MPN has been used to assess viability after treatment of cultured plankton (Oemcke and Van Leeuwen 2005), but to the best of our knowledge, the application of the SDC-MPN method to enumerate total viable phytoplankton after ballast water treatment of natural assemblages has not been examined directly in the scientific literature.

## The SDC-MPN method for total viable phytoplankton

The principles of the SDC-MPN method (Fig. 1) are straightforward, and calculation of MPN from the scores of a dilution series has been examined thoroughly over the years (e.g., Hurley and Roscoe 1983). Agreeing with Cochran’s (1950) opinion that it is more important to be clear about the method’s assumptions than about the details of the mathematics (which are not controversial), we address here the assumptions of SDC-MPN specifically as they apply to the enumeration of viable phytoplankton in natural communities of plankton subjected to BWT, focusing our discussion on the errors that result when these assumptions are not satisfied. We also discuss briefly the application of the method to enumerate only phytoplankton in the 10–50 μm size range and the enumeration of other viable protists.

### False positives and false negatives

**Fig. 2:**
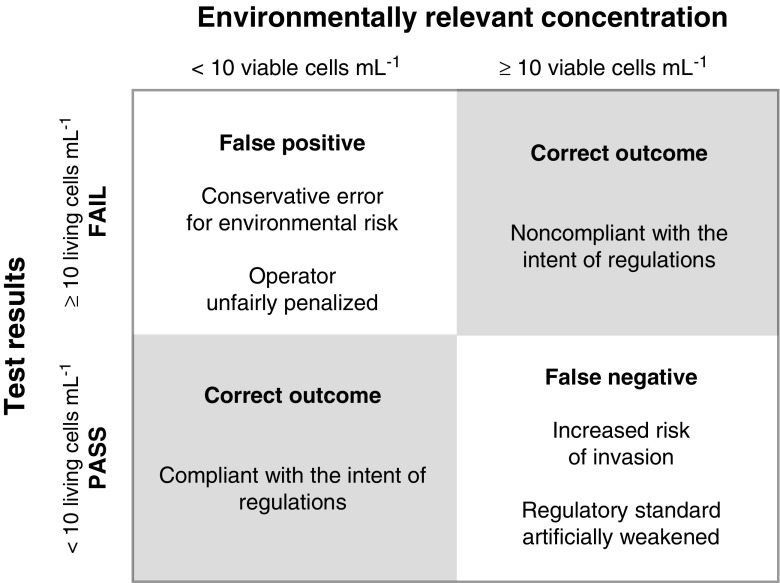
Potential results of tests to enumerate the concentration of living plankton in the 10–50 μm size range in ballast water discharge—i.e., regulations as they stand—assessed in the context of the intent of regulation as reflected in the postulate of equivalent protection (see text). Viable cells mL^−1^ in discharge are referred to as environmentally relevant concentrations to reflect the risk of invasion. Test results are in living cells mL^−1^, consistent with existing USGS and IMO live/dead testing criteria. Like the US Environmental Protection Agency’s Environmental Technology Verification Program (ETV 2010), we assume the perspective of a regulator committed to environmental protection and classify a false positive result as a conservative error, even though it will unfairly penalize the BWMS manufacturer or ship operator. Figure is adapted from Frazier et al. (2013)

An illustration of the potential outcomes of a live/dead test for BWT compliance (Fig. 2) guides our discussion of potential errors in assessing the invasive potential of phytoplankton in ballast water discharge. Embracing the intention of ballast water regulation—to prevent the introduction of propagules of invasive organisms (see, e.g., Reavie et al. 2010)—we apply the postulate of equivalent protection and identify the number of viable cells mL^−1^ in ballast water discharge as the “environmentally relevant concentration” for assessing compliance with the intent of regulations. To reflect existing regulatory criteria and testing methods (e.g., vital stains, see Introduction), test results are represented as living cells mL^−1^. In this context, two types of error are possible:

- False positive—Cells that are not viable (i.e., incapable of reproduction) are classified as living and thus potentially invasive: This error (statistically classified as type I, Frazier et al. 2013) can lead to regulatory failure of a BWT system that is compliant with the intention of regulations. The error is conservative with respect to environmental protection (ETV 2010; US Coast Guard 2012).
- False negative—Viable (therefore potentially invasive and by definition living) cells are incorrectly classified as dead and incapable of invasion (statistically classified as a type II error, Frazier et al. 2013): If this error leads to acceptance of BWT systems that exceed discharge limits, regulatory standards are artificially weakened and the risk of species invasions is increased.

Clearly, the principal concern in BWT testing is the potential for false negatives, because protection of the environment is at stake. False positives are also failures of the test, but when we discuss assumptions or experimental biases that could lead to false positives, we refer to the affected results as “conservative” because the risk of invasion is not increased (Fig. 2). But, it should be recognized that consistently false positives could lead to unwarranted exclusion of particular BWT systems or a technology (e.g., UV disinfection, an effective water treatment technology that does not require chemical biocides), and this might indirectly affect environmental protection by compromising the BWT sector’s abilities to provide innovative and economical treatment solutions.

The classification of errors and their implications are the same as in Fig. 2 when results for individual tubes in the SDC-MPN method are considered, but there is no complication from the distinction between viability and vitality. There are only two correct outcomes: A tube with one or more viable cells should register growth and a tube with no viable cells should register no growth; the alternatives are false negatives or false positives, respectively. We now discuss sources of these errors in SDC-MPN and their likelihood of affecting the outcome of tests on natural phytoplankton, particularly in the 10–50 μm size range. Interference from, and the enumeration of, viable heterotrophs will be considered peripherally.

### Sources of error

The SDC-MPN method, and other assays based on observing the growth of natural phytoplankton after ballast water treatment, have been identified as being definitive because they assess viability (First and Drake 2013a), but such grow-out assays have been discounted for being time-consuming and thus inappropriate for use in rapid assessment of the efficacy of BWT (Steinberg et al. 2011), for example, in shipboard compliance testing (King and Tamburri 2010) and port state control inspection (Drake et al. 2014). Focusing on the use of SDC-MPN for land-based verification testing to gain type approval, we first identify sources of error in the approach, some of which are closely tied to the time allotted for the assay; ways to characterize and minimize the errors will be discussed in following sections.

#### Failure of viable cells to grow

Potentially, the most potent criticism of SDC-MPN is that because it depends on detecting growth of microbes in culture, it will only account for culturable species (Steinberg et al. 2011), and many microorganisms cannot be cultured (First and Drake 2013a; US Coast Guard 2012). Let us examine the relevance of this criticism to SDC-MPN of phytoplankton.

Consider the term “culturable” when used as a general characterization of a microbe: this is not an inherent property of a species but rather a reflection of the culturist’s ability to provide what the organism needs for it to be maintained indefinitely. Studies of microbial diversity reveal many species or ecotypes that are not in culture, for example, the unicellular diazotrophic cyanobacterium UCYN-A (Zehr et al. 2001; Krupke et al. 2014), marine heterotrophic protists (Heywood et al. 2010), and numerous picoeukaryotes (Shi et al. 2009). But with skill, insight, and diligence, researchers have brought fastidious species or those with special requirements into culture for further study. For example, Rappé et al. (2002) isolated the key bacterial species *Pelagibacter ubique* 12 years after its sequence was determined as SAR11 (Giovannoni et al. 1990 as discussed by Vaulot et al. 2008), and it took years to develop effective procedures for culturing a wide range of isolates of *Prochlorococcus* (reviewed by Moore et al. 2007), the most abundant unicellular cyanobacterium on Earth (Partensky et al. 1999). Notably, reports on microbial biodiversity tend to use the terms “uncultured” (Rodriguez-Martinez et al. 2013) or “uncultivated” (Vaulot et al. 2008), implying no judgment on the culturability of species that have yet to be isolated and grown in culture. In the abstract of their study of the growth on agar of heterotrophic microbes from marine sediment, Kaeberlein et al. (2002) stated without detailed explanation that, “The majority (>99 %) of microorganisms from the environment resist cultivation in the laboratory”; we do not find this generalization to be helpful or well substantiated in assessing the culturability of phytoplankton. While it is true that some plankton in the 10–50 μm size range, such as the obligate kleptochloroplastic dinoflagellate *Dinophysis acuminata*, have required extraordinary efforts to bring them into sustained culture (Park et al. 2006), we were unable to find a body of evidence to support the belief that a majority of phytoplankton in that size class cannot be cultured. Regardless, it is important to remember that the SDC-MPN method requires only that viable cells in the dilution cultures multiply enough for their growth to be detected; they need not be maintained through successive transfers. Addressing directly the assumption that cells that are viable in the sea will grow under the conditions provided in dilution culture, Throndsen (1978) noted that “Some species with special requirements will regularly grow up in dilution cultures though they will not survive subculturing” (p. 218), and he advised that the number of species that would grow would be increased if the dilution media was based on the same water from which the sample was taken. Even *Dinophysis acuta*, an obligate grazer which can only be cultured when supplied with its preferred prey, the ciliate *Myrionecta rubra*, will continue to divide for three to four generations in the absence of its prey (Nielsen et al. 2013).

Although the vast range of environmental requirements of phytoplankton ensures that no one set of conditions can support the growth of all viable cells, we are aware of no evidence to suggest that a large component of viable natural phytoplankton are inherently unable to grow through enough divisions to be detected in a suitably designed dilution culture. We suggest that if a viable phytoplankton cell fails to reproduce enough to be detected in SDC-MPN, the false negative result is better ascribed to unsuitable growth conditions in the assay leading to slow or no growth, rather than to inherent properties of some, or many species. Fastidious, fragile, or finicky phytoplankton species certainly exist, and each species or strain has its own environmental optima and limits. Below, we discuss how to minimize false negatives due to slow growth of phytoplankton in dilution culture.

#### Failure to detect growth of the culture

As explained in the Background section, many studies that employed the SDC-MPN method were directed toward the enumeration and ultimate isolation of coexisting species, in part to describe community structure; the dilution-culture tubes were examined microscopically after incubations of weeks to months. If the intention is solely to enumerate viable phytoplankton, as it is with ballast water testing, there is no need for identification, but growth must be detected reliably even when it starts from one cell in a tube.

When SDC-MPN experiments are conducted on unialgal cultures in the laboratory, the likelihood of false negatives for viability can be reduced to near zero through the application of rigorous and sometimes labor-intensive procedures for reliable detection of growth in the dilution tubes. We discuss these in a study of variability among phytoplankton taxa in viability vs. UVC-dose relationships (H.L. MacIntyre et al., submitted for publication). Recognizing that for practical reasons, routine assessment of BWT on natural phytoplankton will need streamlined procedures, provided associated uncertainties can be constrained, we consider the principal influences on accurate detection of growth: (i) the minimum number of cells that can be detected reliably (*N*_d_, cells tube^−1^); (ii) the time it takes for a dilution culture to reach that threshold (*t*_d_, day); and (iii) when the observations are made (*t*_obs_, day). Assuming for now that growth is exponential at rate *μ* (day^−1^) from the initiation of a dilution culture with *N*_0_ cells tube^−1^, the number of detectable cells is reached at time *t*_d_:

1 $$ {N}_{\mathrm{d}}={N}_0\cdot {e}^{\mu \cdot {t}_{\mathrm{d}}} $$

**Fig. 3:**
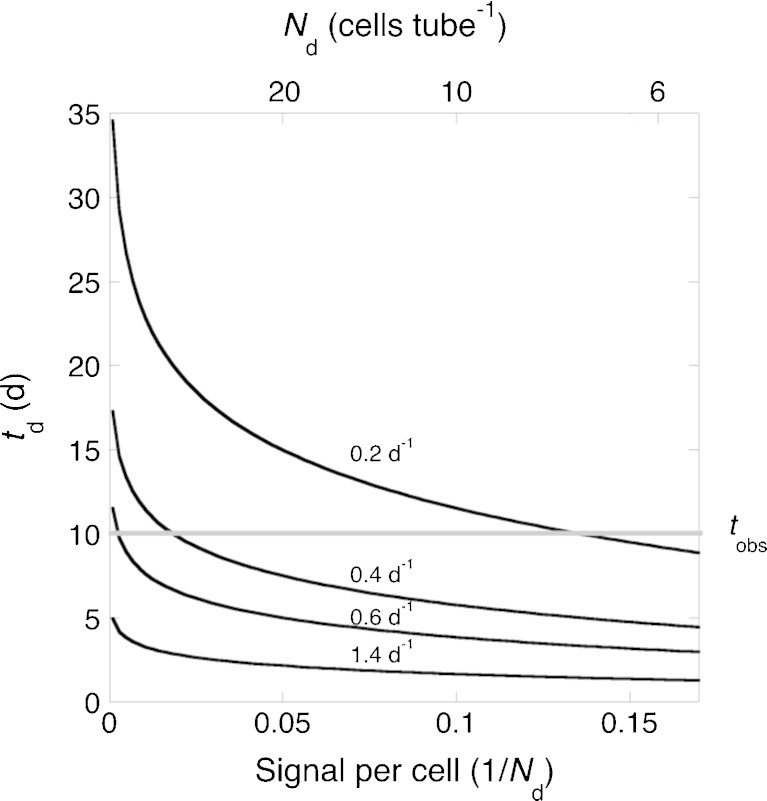
Influence of the detectability and growth rate of cells on the accurate determination of growth in dilution cultures beginning with one cell per tube. The signal per cell is 1/*N*_d_, where *N*_d_ is the minimum number of cells per tube that can be detected reliably and related to growth (e.g., Table 1). The time required for this detection, *t*_d_ (day), is plotted for a range of exponential growth rates. The *gray line* indicates the observation period for the experiment, *t*_obs_ (day)—in this case, 10 days. *Points below the line* represent combinations of growth rate and cell signal that would lead to reliable detection of growth. Slower growing or less effectively detected cells will register growth if *t*_obs_ is increased (raising the *gray line*); the sensitivity of the detection method is increased (moving points to the right along the curves for each growth rate); or conditions are optimized to increase growth rates (cells move to a lower line)

The solution for *t*_d_ is

2 $$ {t}_{\mathrm{d}}=\frac{ \ln \left(\raisebox{1ex}{${N}_{\mathrm{d}}$}\!\left/ \!\raisebox{-1ex}{${N}_0$}\right.\right)}{\mu } $$

which reduces to *t*_d_ = ln(*N*_d_)/*μ* when a culture starts with one viable cell—a straightforward relationship that illustrates the influences of detectability and growth rate on the time required for detection (Fig. 3, Table 1). If growth is delayed by a lag phase (Wood et al. 2004), as might be associated with photorepair (Liebich et al. 2012; Roy 2000), *t*_*d*_ would be incremented by a lag time, *t*_lag_ *.* In either case, if cells are growing in the tube but observations are discontinued prior to *t*_d_, no growth will be detected and a false negative will result. This can be due to slow growth or poor detectability of cells—either because the signal per cell is small (e.g., weakly pigmented phytoplankton) or the instrument is not sufficiently sensitive to detect growth in the time frame of the observations. Remedies include optimizing growth conditions, increasing the sensitivity of detection and extending the period of observations (Fig. 3).

**Table 1:** Time required to detect phytoplankton growth (Eq. 2) in dilution cultures beginning with one viable cell in a culture tube containing a volume of 5 mL, assuming that the lower limit of detection (LLD_0.1_) corresponds to 0.1 mg chlorophyll *a* m^−3^, a concentration that is readily resolved with fluorometers deployed in the open ocean (e.g., Letelier et al. 2004); the corresponding minimum number of cells per tube is *N*_d_

	Dimension (μm)	Chlorophyll quota (pg cell^−1^)	Growth rate (day^−1^)	*N*_d_ (cells tube^−1^)	Time to detection (*t*_d_, day^−1^)
*Gymnodinium vitiligo* ^a^	11	2.31	0.22	216	24.4
*Rhodomonas salina* ^c^	11.6	0.89	0.64	562	9.9
*Thalassiosira weissflogii* ^c^	13.0	3.07	0.83	163	6.1
*Heterosigma akashiwo* ^c^	15.5	2.65	0.56	189	9.4
*Chlamydomonas* sp.^a^	18.6	11.3	0.28	44	13.5
*Gymnodinium sanguineum* ^b^	44	20.4	0.23	9	13.9

The size is minimum dimension (Montagnes et al. 1994) or average diameter (Neale et al. 1998, H.L. MacIntyre et al. unpublished)

^a^Montagnes et al. (1994): 16 °C

^b^Now *Akashiwo sanguinea*. From Neale et al. (1998): 25 °C

^c^H.L. MacIntyre et al. unpublished: 18 °C

#### Competition

It has long been recognized that when SDC-MPN is used to enumerate multiple coexisting species in a natural sample, competition between species of phytoplankton is a concern (Andersen and Throndsen 2003; Throndsen and Kristiansen 1991). For example, Cerino and Zingone (2006) remarked that species that grow in serial dilution cultures often do not survive due to competition. But, competition can only occur when a culture starts with representatives of two or more species in a tube (found at the lower dilutions in a series), and it affects only the estimates of the abundances at the species level; as long as one of the competitors grows to detection, tubes with more than one viable species in the inoculum score positive and the accuracy of SDC-MPN for total viable cells is unaffected. We note that one extreme of competitive exclusion, allelopathic suppression of one species by another through production of growth-inhibiting metabolites, is more likely to occur at higher population densities, when nutrients have been depleted (Granéli and Salomon 2010; Hardison et al. 2013; Tameishi et al. 2009). These conditions are not likely to occur during an SDC-MPN assay.

#### Grazing

Acknowledging that viable heterotrophs in the 10–50 μm size range would have to be enumerated using alternate methods (see, e.g., Zetsche and Meysman 2012; Lighthart 1969), we consider the possible effects of grazing (phagotrophy) on the results of SDC-MPN for viable phytoplankton. A false negative will result if a viable grazer co-occurs with one or more viable phytoplankton and prevents them from growing to detection—an outcome that is not simply predicted given the dependence of grazing rates on prey concentration (Strom et al. 2000) and the artificially low initial concentrations of prey in a dilution series. It is relevant that oceanographers routinely assess microzooplankton grazing rates in the field by progressively diluting natural plankton up to 1:20 with filtered sea water to reduce the encounter frequency between microzooplankton grazers and phytoplankton prey—and thus grazing losses (Landry and Hassett 1982). In addition, the co-occurrence of grazers and phytoplankton becomes increasingly unlikely in the most-diluted tubes. We therefore suggest that grazing is not likely to cause significant numbers of false negatives in SDC-MPN tubes, and when it does, it will not strongly affect the calculated result: The errors will be in the less-diluted series that have a smaller quantitative influence on the estimate of total viable cells. A conservative solution, if practicable, would be to score all tubes containing heterotrophs at *t*_obs_ as positive for phytoplankton growth, thereby ensuring that no viable phytoplankton cell was overlooked due to grazing. This approach would be particularly useful for heterotrophic dinoflagellates, which can be important consumers of diatoms, differ fundamentally from other microzooplankton in their feeding and growth dynamics, and tend to survive well when their food supply is exhausted (Sherr and Sherr 2007).

#### Inclusion of nonregulated organisms

If the method of detection, e.g., in vivo fluorescence (Brand and Guillard 1981b; Wood et al. 2004), is a bulk measurement that does not distinguish cell size, nonregulated phytoplankton <10 μm, or more stringently regulated phytoplankton ≥50 μm, could grow and produce: (i) false positive scores for the 10–50 μm size range if no viable phytoplankton in the range were in the dilution tube or (ii) true positive scores if at least one viable regulated cell was in the tube but was outcompeted. If all tubes with positive scores are scored as such, regardless of cell size, the result is conservative, reducing risk to the environment, but cells <10 μm are generally much more abundant than the larger regulated cells (Marañón 2015), and significant overestimation of the numbers of viable 10–50 μm cells can be expected. The likelihood of error can be reduced greatly by prefiltration to remove larger cells and gentle concentration on a 10-μm mesh to retain cells in the regulated size range (Reavie et al. 2010; First and Drake 2013b).

#### Aggregation of phytoplankton cells

The SDC-MPN method depends on the assumption that organisms are randomly distributed in each tube and evenly distributed between subsamples (Cochran 1950). The assumption will be violated, and numbers of viable cells will be underestimated, if phytoplankton grow in colonies that cannot be broken up without compromising viability or if individual cells or colonies collect in aggregates (Revelante and Gilmartin 1991) that are incompletely disrupted during the sampling and dilution process. Thorough but relatively gentle mixing (e.g., 100 inversions, Andersen and Throndsen 2003) is recommended prior to any subsampling, but harsher methods such as sonication or heating followed by vortex mixing (see Humphries and Widjaja 1979) have been used to break up colonies of cyanobacteria. Even though conditions during ballast water transfers and treatment are anything but placid, harsh disruption of aggregates would likely introduce unacceptable uncertainty into tests of viability after dilution. The importance of aggregation in samples can be assessed with microscopic examination or by testing for uniformity of repeated bulk measurements (e.g., chlorophyll *a*) made on subsamples.

Colonies such as diatom chains would be expected to persist, and they would complicate SDC-MPN because in dilution, they are distributed as entities (after Reavie et al. 2010) that contain more than one cell. The statistics of MPN would estimate the number of viable entities, but this would be less than the number of viable cells. If a colony of *x* viable cells released into a foreign environment acts more like one propagule rather than *x* propagules, however, the error would be more numerical than ecological, but the numerical result counts for regulation.

## Toward best practices for minimizing error

We suggest that competition and grazing are not important sources of error when SDC-MPN is used to enumerate total viable phytoplankton. The other sources of error might not be totally eliminated, but they could be assessed and minimized wherever practically possible, thereby reducing the uncertainty of SDC-MPN counts of total viable cells for regulation of BWT.

### Optimizing growth conditions

First and foremost, the accuracy of the SDC-MPN method depends on its ability to provide all viable phytoplankton cells with conditions—light, temperature, and chemical milieu—to support growth in highly dilute culture, and the growth must be rapid enough to ensure that cell numbers can increase from a single cell in a tube to the level of reliable detection during the observation period (Fig. 3, Table 1). Given that differentiation between species in growth responses to light, nutrients, and temperature is a foundation of diversity in phytoplankton (Follows et al. 2007; Johnson et al. 2006) [but see Cullen and MacIntyre (1998) for discussions of behavior and physiological plasticity and Verity and Smetacek (1996) for top-down control], it is self-evident that no one set of conditions during SDC-MPN can ensure optimal growth of all viable phytoplankton in a sample. But, decades of research and experience can inform the process of choosing conditions in SDC grow-out so that the exclusion of species due to unsuitable growth conditions is minimized.

A good place to begin is with advice from experts who have used SDC to both enumerate and isolate phytoplankton for culturing. Concluding suggestions from Andersen and Throndsen (2003) are succinct and exemplary: “The success or failure of the method depends on (a) the cleanliness of the equipment; (b) the suitability of the growth medium; and (c) the external culture conditions (temperature and light)” (p. 128).

#### Growth media

Cleanliness is a prerequisite for reliable culturing of phytoplankton in dilution culture (discussed by Guillard 2005), and sterile technique is required to avoid contamination (Kawachi and Noël 2005). A variety of growth media can be used, depending on the application (Andersen and Kawachi 2005). Guillard and Morton (2003) provide specific advice on the growth medium, explaining that the purpose of nutrient enrichments is to provide scarce materials to phytoplankton in usable forms at levels that are neither toxic nor limiting for growth rate. For general enrichment cultures, they specify nutrient additions not to exceed concentrations that are lower than for typical culture media (compare their recommendations with recipes from Appendix A in Andersen 2005), and they include nitrate, ammonium, and urea as nitrogen sources, thereby accommodating species that do not utilize nitrate, e.g., many strains of *Prochlorococcus* (Moore et al. 2002). Throndsen (1978) recommends a modified Erd-Schreiber medium (pasteurized) that has higher concentrations of nutrients and includes soil extract; Guillard and Morton (2003) recognize soil extract as often being beneficial, and they provide a recipe. Andersen and Throndsen (2003) mention that dilutions prepared with water from the sampling site yield cultures with higher species diversity than premade growth medium, implying that more species are able to grow in water from their source. In the absence of a comprehensive review that would be beyond the scope of this discussion, it seems reasonable to adopt Andersen and Throndsen’s (2003) suggestion that the media for serial dilution culture should be prepared with water sampled together with the inoculum, with nutrient enrichments that are high enough to ensure growth past the point of detection but lower than in conventional growth media (see, e.g., Guillard and Morton 2003; Andersen and Kawachi 2005). Although we expect no one recipe to ensure optimal growth for a maximum number of species in all BWT conditions, we offer provisional guidance in Table 2, based on the expert advice cited above. As with other recommended practices, the way to evaluate them is through experimental comparisons.

**Table 2:** Provisional recommended practices for SDC-MPN on natural phytoplankton assemblages in land-based testing for BWMS type approval, suitable for being evaluated through systematic comparisons

	Standard	Optimized
Growth medium		
Source water	Filtered water from the original sample	
Nutrient enrichment (options)	Reduced concentrations from typical growth media (include soil extract, multiple nitrogen sources)	
Sterilization	Filter sterilization, pasteurization	
Temperature (°C)	*T*_in situ_ or ${\overline{T}}_{\mathrm{in}\ \mathrm{situ}}$	${\overline{T}}_{\mathrm{in}\ \mathrm{situ}}$ + 5 °C for ${\overline{T}}_{\mathrm{in}\ \mathrm{situ}}$ ≤20 °C decreasing linearly to 0 offset at 28 °C
Irradiance (μmol photons m^−2^ s^−1^)	Light/dark cycle ~10 % *Ē*_0 cf_ (~60–200)	Longer light period (e.g., 16 h) 18 % *Ē*_0_ or $82+\left(4.7\cdot {\overline{T}}_{\mathrm{in}\ \mathrm{situ}}\right)+0.068\cdot \left({\left({\overline{T}}_{\mathrm{in}\ \mathrm{situ}}\right)}^2\right)$

Standard conditions are based on recommendations in the literature, intended to maximize the number of species that will grow. The optimized conditions proposed here are intended to support enhanced growth rates without significant reductions in the number of species that will grow, thereby shortening the time required to detect viable cells in an SDC-MPN assay and minimizing false negatives due to slowly growing isolates. Ambient water temperature is *T*_in situ_ (°C); the climatological average is ${\overline{T}}_{\mathrm{in}\ \mathrm{situ}}$; climatological cloud-free midday irradiance is *Ē*_0 cf_ (μmol photons m^−2^ s^−1^ PAR); and climatological midday irradiance is *Ē*_0_

Options for sterilization of the media are comprehensively reviewed by Kawachi and Noël (2005); they include filter sterilization, which will retain potentially important heat-labile compounds such as vitamins in the source water, but which will allow viruses to pass. Viruses and even proteins can be removed by tangential-flow filtration (van Reis et al. 1999), but the benefit of the removal of the viruses might be outweighed by the removal of nutrients essential for growth of auxotrophic species. However, as with grazing, the influence of viruses is expected to be reduced in the most dilute samples (Andersen and Throndsen 2003).

#### Temperature

It is a central tenet of plankton ecology that in the absence of nutrient limitation, light and temperature are the principal influences on the growth rates of phytoplankton (Cullen et al. 1993; Yoder 1979). Through a combination of competition and the constraints of absolute environmental tolerances, light and temperature regimes select for phytoplankton species and ecotypes, explaining dominant patterns in their distributions (Follows et al. 2007; Johnson et al. 2006), although these are, of course, also influenced strongly by nutrients, food web interactions, and physical processes such as mixing (Follows and Dutkiewicz 2011; Cullen et al. 2002).

Generally, discussions of the incubation temperature for dilution culture have focused on controlling it to avoid harmful or lethal variation, rather than choosing a temperature to optimize growth rate (Knight-Jones 1951; Guillard and Morton 2003; Andersen and Throndsen 2003). Considering that the duration of SDC-MPN—that is, *t*_obs_ in Eq. 2—should for practical reasons be no longer than necessary, there are good reasons to optimize growth rate to minimize the time of detection, *t*_d_, for as many species as possible (see Fig. 3). Choice of an incubation temperature is an important factor.

**Fig. 4:**
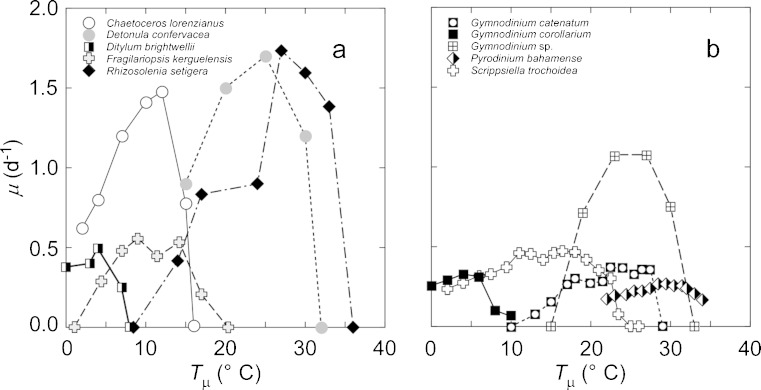
Specific growth rates, *μ* (day^−1^), of cultures of **a** diatoms and **b** dinoflagellates as a function of growth temperature, *T*_μ_ (°C). Data sources: *Chaetoceros lorenzianus* (Hulburt 1982); *Detonula confervacea* (Smayda 1969); *Ditylum brightwellii* (Paasche 1968); *Fragilariopsis kerguelensis* (Fiala and Oriol 1990); *Gymnodinium catenatum* (Bravo and Anderson 1994); *G. corollarium* (Sundström et al. 2009); *G*. sp. (Thomas 1966); *Pyrodinium bahamense* (Usup et al. 1994); *Scrippsiella trochoidea* (Binder and Anderson 1987)

The responses of phytoplankton growth rates to temperature (Fig. 4) illustrate well-known features that should be considered: Some species grow more quickly than others; all show increasing growth rate with temperature up to a maximum; some have very broad tolerance ranges while others do not; and the decline of growth rate with increasing temperature above the optimum is sharper than the increase with temperature below it, due primarily to reductions in protein and cytochrome functionality (e.g., Fork et al. 1979; Gao et al. 2000; Nitta et al. 2006).

**Fig. 5:**
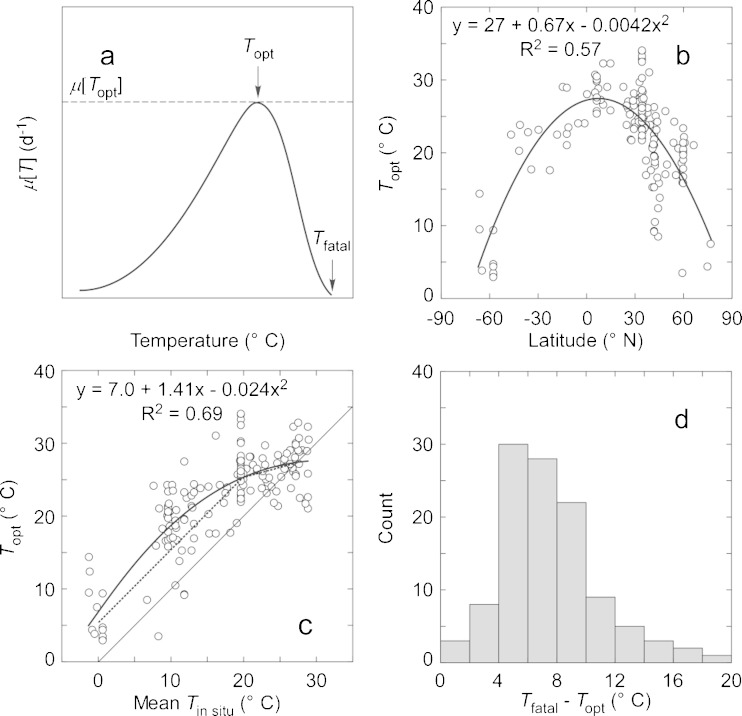
**a** Generalized form of the relationship between growth rate (*μ*(*T*), day^−1^) and temperature (Eq. 3, based on Blanchard et al. 1996), showing the optimal temperature (*T*_opt_) at which growth rate is maximal and the fatal temperature (*T*_fatal_) at which growth is completely abolished. **b** Latitudinal variation in optimal growth temperature in 163 cultures of estuarine and marine phytoplankton obtained by re-fitting data compiled by Thomas et al. (2012) to Eq. 3. The data have been fit to a second-order polynomial (*heavy line and equation*). **c** The same estimates of *T*_opt_ as a function of the mean annual temperature at their isolation locations (from Thomas et al. 2012). The *straight solid line* shows a 1:1 relationship; the *curved line* is a fit to a second-order polynomial, and the *dotted line* represents a provisional recommendation for incubation temperature during serial dilution culture (Table 2). **d** Frequency distribution of the temperature difference between the fatal and optimal temperatures for the cultures in **b**. Only fits in which the error on *T*_fatal_ was less than 20 % of the estimate (*n* = 111) are included to avoid bias from fits in which the estimate of *T*_fatal_ is uncertain due to extrapolation. The mean difference is 7.5 °C, and the median is 7.1 °C

The response of phytoplankton growth rate to temperature can be generalized by fitting it to the functional form of a model describing the short-term influence of temperature on the photosynthetic capacity of natural benthic diatoms (Blanchard et al. 1996):

3 $$ {\mu}_{\max }(T)={\mu}_{\max}\left({T}_{\mathrm{opt}}\right)\cdot {\left(\frac{T_{\mathrm{fatal}}-T}{T_{\mathrm{fatal}}-{T}_{\mathrm{opt}}}\right)}^{\beta}\cdot \exp \left[-\beta \left(\frac{T_{\mathrm{fatal}}-T}{T_{\mathrm{fatal}}-{T}_{\mathrm{opt}}}-1\right)\right] $$

where *μ*_max_(*T*) (day^−1^) is the maximum growth rate at temperature *T* (°C), i.e., not limited by light or nutrients, *T*_opt_ (°C) is the temperature at which growth rate is maximal at the rate *μ*_max_ (*T*_opt_) (day^−1^), *T*_fatal_ (°C) is the temperature at which growth rate declines to zero, and *β* is a dimensionless shape factor (Fig. 5a).

To describe general trends in the temperature responses of cultured phytoplankton with explicit calculation of fatal temperature, we fit to Eq. 3 data on growth rates of cultures of marine and estuarine phytoplankton compiled by Thomas et al. (2012). The equations were fitted using a Levenberg-Marquardt method in Kaleidagraph 4.5 (Synergy Software). Because Eq. 3 contains four parameters, only studies with five or more data points were included. As in the Thomas et al. (2012) analysis that used a different temperature function to estimate *T*_opt_ (i.e., Norberg 2004), the resulting estimates of optimal growth temperatures for 163 cultures had a significant relationship with the latitude of isolation (Fig. 5b), consistent with annual mean temperature in situ being an important selective factor that is reflected in *T*_opt_ (Fig. 5c). Note the dispersion of data around the central tendencies: this may reflect a link between seasonal variations in temperature and community composition that cannot be revealed through the analysis. As concluded by Thomas et al. (2012), competition between species with comparable growth rates will tend to favor taxa for which *T*_opt_ is above, but close to the in situ temperature, possibly subject to an inherent maximum near 30 °C. The implication for BWT testing is that if the temperature of an SDC-MPN assay is chosen to optimize growth for the indigenous community, the test is unlikely to select against potentially successful invaders because they are likely to share temperature optima with established members of phytoplankton communities.

We therefore suggest that SDC-MPN assays be conducted at temperatures above ambient, thereby increasing growth rates and minimizing time to detection (Table 2). The relationship between *T*_opt_ and the annual mean temperature at the location of isolation suggests that growth rates would be higher at temperatures about 5 °C above ambient up to about 20 °C for the in situ temperature, decreasing to no offset at 28 °C, as illustrated with the dotted line in Fig. 5c. For the growth data analyzed in Fig. 5, increasing the temperature to *T*_opt_ from 5 °C below it results in an increase in specific growth rate of 5–660 % (median 26 %, *n* = 163). The variability arises because of differences in the steepness of the relationship at the growth optimum (see Fig. 4). The increases in growth rates translate to reductions of 5–85 % (median 21 %) in the time required to detect growth (Eq. 2). However, it is important not to impose thermal stress by approaching closely or exceeding the fatal temperatures for phytoplankton in the samples. The frequency distribution of *T*_fatal_ − *T*_opt_ (Fig. 5d)—a measure of the margin for error in overestimating *T*_opt_—suggests that the risk of approaching *T*_fatal_ when targeting *T*_opt_ for incubation temperature is low until the incubation temperature exceeds *T*_opt_ of a species by about 5 °C. The validity of this inference can be tested through systematic comparison in parallel of SDC-MPN experiments conducted at the in situ temperature and at the purported optimized temperature.

#### Light

For SDC-MPN of phytoplankton collected from surface waters, recommendations based on experience suggest that incubation light levels, *E*_inc_, should be about 10 % of full daylight (Andersen and Throndsen 2003). This corresponds roughly to 60–200 μmol photons m^−2^ s^−1^ for the winter vs. summer solstice at 45° latitude (photosynthetically available radiation, clear-sky calculations based on Bird and Riordan 1986), similar to the range suggested by Guillard and Morton (2003), who add that fluorescent bulbs remain the light source of choice and caution that some algae require a dark period to survive (Brand and Guillard 1981a), so light–dark incubation conditions should be used (Table 2).

**Fig. 6:**
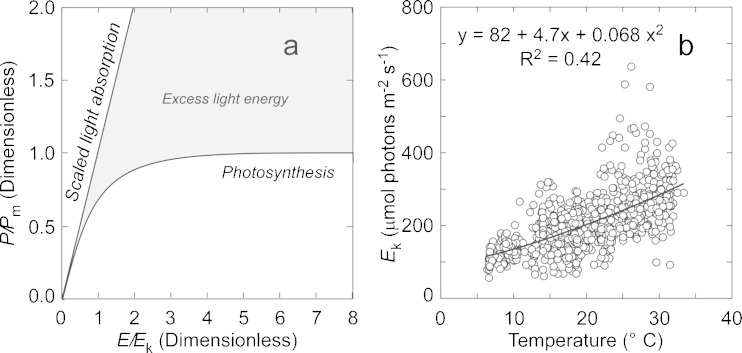
**a** A generalized photosynthesis-irradiance (*P* vs. *E*) curve, shown in dimensionless form: Photosynthesis has been scaled to its light-saturated rate, *P*_m_, and irradiance has been scaled to the light-saturation parameter, *E*_k_. The scaled rate of light absorption is also shown: The *shaded area* between the two curves represents light energy that is absorbed by the cell but not used in photosynthesis. This must be dissipated to avoid oxidative stress; incubating cells at irradiances ≫*E*_k_ is likely to result in reduction in photosynthetic rate (i.e., photoinhibition) and growth rate. **b** The temperature-dependence of *E*_k_ in 820 *P* vs. *E* curves from estuarine, neritic, and oceanic waters off North America (H.L. MacIntyre et al., unpublished data). The data have been fit (*heavy line and equation*) to a second-order polynomial. Note the dispersion of data around the central tendency: 94 % are between 0.5 and 2x of the fitted value. Incubating samples at the irradiance predicted by the equation (Table 2) should ensure than the overwhelming majority of samples are exposed to irradiances that are between 0.5 and 2x *E*_k_, a range over which photosynthesis achieves 14–61 % of the light-saturated rate and over which oxidative stress is minimal. Growth rates are expected to scale directly with the achieved photosynthetic rate

In an attempt to explore the optimization of light level for SDC-MPN, we consider the relationship between photosynthesis and irradiance (*P* vs. *E*), which is not synonymous with growth rate vs. irradiance but which is intimately linked to it (Geider et al. 1998; Cullen 1990). It is described by a maximum rate, *P*_m_ (e.g., mg C m^−3^ h^−1^), and the light saturation parameter, *E*_k_ (μmol photons m^−2^ s^−1^) (Talling 1957). As ambient irradiance exceeds *E*_k_, photosynthetic rate is saturated, absorbed light energy must be otherwise dissipated, and phytoplankton are subject to stress that can lead to the inhibition of photosynthesis and a decrease of growth rate (Fig. 6a; Baroli and Melis 1996; Ritchie and Larkum 2012); in turn, it is expected that incubation of phytoplankton at the *E*_k_ for *P* vs. *E* should support relatively high growth rates with little stress. Since *E*_k_ is an essential reflection of physiological and taxonomic responses of phytoplankton to irradiance—it expresses both phenotypic and genotypic variability—it is rightly called the photoacclimation or photoadaptation parameter and it covaries with the irradiance at which phytoplankton are grown (Bannister and Laws 1980; MacIntyre et al. 2002). A review is well beyond the scope of this discussion, but in the context of SDC-MPN, it is relevant to observe that the ratio of surface irradiance to *E*_k_ is a key metric in models of primary productivity for remote sensing (Behrenfeld and Falkowski 1997; Platt and Sathyendranath 1993; Cullen et al. 2012). Platt and Sathyendranath (1993) found that about half of the variability of *E*_k_ for the top 40 m of the water column could be explained by variations of surface irradiance: The best estimate of the ratio of *E*_k_ to the midday irradiance averaged over 3 days was 0.18 (with individual observations typically in the range of 0.13–0.20), which leads us to recommend *E*_inc_ = *E*_k_ of 18 % of the average midday irradiance at the surface (Table 2), corresponding roughly to 100–350 μm photons m^−2^ s^−1^ for the winter- vs. summer solstice at 45° latitude as referenced above.

Temperature also influences *E*_k_ through its effect on enzymatic rates (Hancke et al. 2008; Hikosaka et al. 2006). Thus, for a given irradiance and nutritional status, models of photosynthesis and growth specify *E*_k_ and growth rates increasing exponentially with temperature (Lima and Doney 2004; Geider et al. 1998). Because on a global scale, temperature tends to covary with midday irradiance, relationships between *E*_k_ and surface irradiance such as that presented by Platt and Sathyendranath (1993) would also include effects of temperature that are more contributory than confounding (cf. Harrison and Platt 1980, 1986). An extensive set of 820 *P* vs. *E* experiments for which temperature (but not average midday irradiance) was measured shows that, consistent with predictions based on light alone, *E*_k_ varies from about 100–350 μm photons m^−2^ s^−1^ over the range from cold, low-light to warm, high-light waters (Fig. 6b).

Our analysis suggests that choosing *E*_inc_ either on the basis of temperature or a measure of midday irradiance (Table 2) will yield estimates with similar ranges, and either would likely support more rapid growth of phytoplankton than incubation at lower irradiance, perhaps faster yet if long day lengths are applied. The effectiveness of optimized conditions would have to be tested.

### Assessing the effects of culture conditions

The intention of optimizing growth conditions is to minimize false negatives due to undetected viable cells, thus maximizing the estimate of total viable phytoplankton. Benign conditions could also favor photorepair of DNA damage (Boelen et al. 2001; Roy 2000) and photosynthetic systems (Tyystjärvi 2008; Campbell et al. 2013) that might not have occurred in nature, leading to more conservative results.

The most straightforward way to assess the suitability of any experimental regime for SDC-MPN is to compare it to others. Assuming for now that the influence of nonregulated plankton (particularly, rapidly growing phytoplankton <10 μm) is minimized or accounted for, the higher counts of viable cells from the same initial sample should be considered more accurate. Two types of comparison can be conducted: parallel and serial.

#### Parallel experiments to test optimization procedures

Experienced practitioners of dilution culture have suggested growth conditions that are intended to maximize the number of species that will grow, not necessarily how fast they grow. Recognizing that because of expense, time is of the essence in the routine application of SDC-MPN for assessment of ballast water treatment, and that faster growth permits shorter observation periods (Eq. 2 and Fig. 3), we present provisional guidance for optimizing growth conditions, including choices of incubation temperature and irradiance higher than the conventional in situ temperature and ~10 % surface irradiance (Table 2). The merit of these suggestions can be assessed by conducting experiments in parallel on the same initial samples in which results for optimized temperature and irradiance are compared to those for conventional conditions.

No significant difference between results for the two growth conditions would suggest that growth rates had little influence on results, for example if *t*_obs_ was long enough to detect even the slower growing cells in conventional conditions. Unfortunately, opposing influences of the changes in light and temperature on growth might produce a similar result, so it would be difficult to draw conclusions from this comparison alone. Uncertainties associated with possible light-temperature interactions could be explored using repeated applications of a 2 × 2 light-temperature design. This would require considerable effort that might be impractical for routine testing programs but would be highly valuable in an effort to characterize rigorously the uncertainties in SDC-MPN and the effectiveness of optimization procedures.

If counts are systematically higher for optimized conditions, it suggests that growth rate influences results over the observation period so that optimized growth conditions lead to more accurate counts. The result does not reveal how many slow-growing species in dilution culture tubes remain undetected under optimal conditions, however. That can be addressed by making repeated observations during the observation period and extending it if necessary, as described below.

#### Serial sampling to assess false negative results

Repeated observations during the grow-out period, for example using nondestructive measurement of in vivo chlorophyll fluorescence (Brand and Guillard 1981b; Wood et al. 2004), can obviate uncertainties associated with cultures that grow but then decline below the detection limit prior to *t*_obs_ (H.L. MacIntyre et al., submitted for publication), and they can provide greater confidence than a single reading above the detection limit that growth of a culture has indeed occurred. But more importantly, perhaps, sequential observations reveal the rate at which new—i.e., slower growing and/or smaller signal-per-cell—cultures are being recruited into the MPN score sheet, thereby increasing the most probable count of total viable cells. Scores that continue to rise day by day (i.e., the consequences of literally raising the *t*_d_ bar in Fig. 3) indicate that the observation period should be extended, whereas little change with time suggests that an asymptote has been reached and the observation period is long enough to ensure stable results. Even one extra observation prior to a terminal assessment at *t*_obs_, made routinely and analyzed systematically, should provide a strong indication of the influence of the choice of observation period on the accuracy of the results.

#### What about the cells that do not grow?

If sequential MPN estimates indicate that a steady maximum is consistently reached, we can infer that the observation period is long enough to detect most of the viable cells that will grow in dilution culture. But what of “SDC ungrowables”—viable cells that grow very slowly or not at all: Are they likely to be a significant source of false negatives? As argued above, Throndsen (1978) observed that some species with special requirements will regularly grow in dilution culture although they will not survive subculturing, and we were unable to find strong evidence for inherent unculturability of many species of phytoplankton. Even so, errors in SDC-MPN due to poor or no growth of viable cells are certainly possible and should be addressed. One approach is to compare SDC-MPN estimates of total viable cells with enumerations of total cells using microscopy or flow cytometry, assuming that all the conventionally enumerated cells are viable and keeping in mind that some species are damaged by common fixatives (Kugrens and Lee 1987). Because intact but nonviable cells are expected after UV treatment, the comparison is appropriate only for untreated controls. Agreement between counts of total cells and SDC-MPN estimates of viable cells, for example as reported by First and Drake (2013b), would suggest that the most abundant viable cells grew in dilution culture and so-called unculturability of species was not a significant problem in that experiment. But, if the estimates of viable cells are significantly lower than counts of total cells, the discrepancy could be due to (i) false negatives in SDC-MPN, the major concern, or (ii) the enumeration of intact cells that are not viable. The latter possibility is suggested by the observation of intact but metabolically inactive cells in early summer assemblages in Lake Rotorua (Paerl 1978) and Blanes Bay (Agustí and Sánchez 2002). In the context of regulation and in the absence of other measures of vitality, the false-negative interpretation is conservative and could be used to establish a measure of the type II error associated with viable cells that do not grow.

The comparison of viable cells vs. total cells in untreated controls puts bounds on the uncertainty of the SDC-MPN method for assessing the effectiveness of BWT, but only to the extent that the influence of SDC-ungrowable species on assay results is the same or less in treated samples as in the controls. The requirement would be violated under a hypothetical scenario that there is no reason to expect a priori: SDC-ungrowable species which (i) are rare compared with dominant species and thus do not significantly influence the enumeration of viable cells in untreated controls but (ii) are also very resistant to treatment compared to the majority of cells so that they represent a significant proportion of viable cells after treatment. The comparison of viable vs. total cells in the untreated control would suggest no problem with the SDC-MPN method, but the disproportionate survival of these rare, resistant, and ungrowable cells would lead to an SDC-MPN underestimation of viable cells after treatment proportional to their abundance relative to surviving growable cells. The error would be relevant to regulation only if the concentrations of surviving, originally rare ungrowables were close to or higher than the regulatory limit of 10 cells mL^−1^.

Our scenario, presented for completeness, might be highly unlikely, but it should be considered. Hypothetically, the problem could be addressed by identifying species in dilution culture grow-outs from untreated plankton communities to see which consistently fail to grow, and then examining culture-independent single-cell measures of their susceptibility to treatment [e.g., pyrimidine dimers, Buma et al. (1995) or other molecular markers to be developed]. But, it should be remembered that competition will influence the results of SDC (Throndsen and Kristiansen 1991; Cerino and Zingone 2006) in all but the extreme dilutions (from which rare species are excluded). Although it would be possible to document rare species that do grow, thereby reducing concern about their complicity false-negative errors, there is as yet no straightforward way to document rare species that do not grow well in dilution culture: failure to detect them in mixed-species cultures is not conclusive evidence of their inability to grow to detection on their own.

We conclude that uncertainties associated with poor growth in dilution culture are unlikely to be fully resolved, although agreement between enumerations of viable cells and counts of total cells would indicate that the errors are not large compared to uncertainties in other metrics of phytoplankton or their activities, discussed below. The potential problem of poor growth in dilution culture has another facet: if conditions are chosen to favor local and regional conditions as suggested here, the species that do not grow well in the assay are demonstrating reduced fitness for invasion compared to competitors that do. It follows that false negatives for phytoplankton that are poorly adapted to locally optimized growth conditions represent a reduced environmental risk compared with underestimation of viable cells from more fit species.

### Quantifying other plankton

The SDC-MPN method as described here is designed for plankton that grow well as photoautotrophs, but regulations apply to all plankton. Alternate approaches are required to enumerate resting stages (Gregg and Hallegraeff 2007) and heterotrophs (Lighthart 1969) in the 10–50 μm size range. If methods for enumerating viable heterotrophs as defined here are not practical for application in routine testing, direct counts of living cells could be used (ETV 2010), probably leading to false positives for UV treatment of heterotrophs.

## Putting errors in context

The SDC-MPN method will never be exact, but few if any techniques for quantifying phytoplankton or their activities are. It is well established that the relationship between in vivo fluorescence and chlorophyll *a* is highly variable, as is the ratio of chlorophyll to phytoplankton biomass (Cullen 1982). Estimates of phytoplankton biomass from space (e.g., Boyce et al. 2010) add yet another level of error that compounds the uncertainty in estimates of primary productivity (Saba et al. 2010; Friedrichs et al. 2009), models of which are grounded in measurements of photosynthesis that are also subject to significant variability due to methods and interpretations (Richardson 1991; Cullen 2001). Yet, there is no question that measurements of chlorophyll, in vivo fluorescence, and primary productivity, along with estimates of phytoplankton biomass and productivity from space, have added immeasurably to our knowledge of planktonic processes, despite inaccuracies inherent in each.

Estimates of phytoplankton vitality as assessed by the vital stain fluorescein diacetate—one of the stains recommended by the ETV panel (EPA 2010) for verifying treatment efficacy—are subject to significant uncertainties from inter- and intra-specific variability in stain uptake (Selvin et al. 1988; Agustí and Sánchez 2002; Garvey et al. 2007; Peperzak and Brussaard 2011). The method has been validated with natural populations by comparison with an enzymic digestion test (Agustí and Sánchez 2002) and by comparing staining in control (live) and heat- or cold-treated samples (Steinberg et al. 2011), but a systematic and quantitative evaluation of false positive and false negative errors across species and growth conditions has not been published; our results on laboratory cultures suggest that the errors can be large (H.L. MacIntyre et al., in prep.). It follows that estimates of total viable phytoplankton from SDC-MPN, though subject to errors described here, could potentially serve an important role as an alternative technique in BWT verification for type approval (see ETV 2010) if uncertainties in the method can be adequately constrained and shown to be acceptable in comparison with other techniques.

## Alternate assays of viability

Growth is the only direct measure of viability, and for ballast water treatment with UV, viability, as compared to vitality, is the only accurate measure of invasive potential. Further, the viability criterion is suitable for assessing other treatment technologies if it is practicable, because cells that have been killed outright are also nonviable. In principle, the SDC-MPN method is the gold standard for enumerating viable cells, but as we have shown, its application for use on natural samples introduces uncertainties that can be minimized but not entirely eliminated. Regardless, the SDC-MPN method can never be used for rapid assays (e.g., minutes, First and Drake 2013b) that are preferred for any BWT testing procedure and required for applications such as routine monitoring of system performance (King and Tamburri 2010). Proxy measurements of both vitality and viability are needed (First and Drake 2013b; Reavie et al. 2010; Steinberg et al. 2012), but they must be validated. Given the live/dead/viable issue with UV radiation, we suggest that proxies must ultimately be tested against viability. In our experience, the SDC-MPN method is well suited for quantitative and accurate implementation with cultures of phytoplankton (MacIntyre et al., submitted for publication, Oemcke and Van Leeuwen 2005), so with appropriate testing and evaluation methodology, it should be possible to develop rapid assays of viability that are rigorously validated through comparisons with direct measurements of survival and growth after treatment.

## Summary and conclusions

This review provides the foundations for the following statements about the use of the SDC-MPN method for enumerating viable phytoplankton in the testing of ballast water management systems:

1. *Postulate of equivalent protection*: Because neither a dead organism nor a nonreproductive organism can propagate after discharge from ships’ ballast, discharge criteria based on vitality (live/dead) and viability (the ability to reproduce) are equally protective of coastal environments.
2. It follows that the concentration of viable phytoplankton cells, as compared to living cells, could serve as an alternative measure for regulating ballast water discharge from any type of management system—if it is established that viable cells in natural phytoplankton samples can be reliably enumerated after BWT.
3. Since UV renders phytoplankton nonreproductive and thus harmless without killing them outright, the effectiveness of ballast water treatment with UV for protecting coastal environments can be assessed accurately only with measures of viability, not vital stains.
4. The SDC-MPN assay enumerates viable phytoplankton. It has been used for more than 50 years in the context of phytoplankton ecology, generally to identify and count phytoplankton species that cannot be preserved for conventional microscopic analysis. To the best of our knowledge, the use of SDC-MPN to enumerate total viable phytoplankton in a natural sample without regard to species composition is a new application emerging from its potential utility in testing BWT systems.
5. When the sole objective is to enumerate total viable cells, the accuracy of SDC-MPN depends primarily on its ability to detect growth in culture tubes starting with one or more viable cells. Concerns about SDC-MPN on natural communities can be addressed. Although some species of phytoplankton are difficult to bring into persistent culture, many are expected to grow in a first-round dilution culture for SDC-MPN if conditions are suitable; this expectation can be tested. Competition between phytoplankton species in dilution cultures is irrelevant as long as the winner is detectable. Grazing is expected to have a relatively small influence on the outcome of SDC-MPN, and conservative results can be obtained if it is practicable to detect grazers in tubes and score them as positive for a viable phytoplankton cell. Recovery from damage during treatment, e.g., photorepair, is not a complication: If the cell survives and grows, it is enumerated as viable, and since growth conditions are optimized, repair is more likely than it might be in nature.
6. The accuracy of SDC-MPN depends on the ability of a single cell in a diluted sample to reproduce through enough generations to be detected reliably—a function of growth rate, signal per cell, detector sensitivity, and the time period of observation. We propose procedures to maximize growth rate and detectability, and thus accuracy of the method, and to estimate uncertainties, which can be reduced through systematic comparisons that we describe.

We conclude that SDC-MPN is potentially an effective method for assessing the viability of phytoplankton after BWT. It has been used in phytoplankton studies for many decades but only recently to enumerate total viable phytoplankton in natural samples. In this application, some sources of error are much less important than previously thought and others can be assessed and minimized, though probably not eliminated. When used on cultures in the laboratory, the SDC-MPN method can serve as the standard for validating proxy measurements of viability that could be useful in shipboard compliance testing and port state control inspection. In all applications, experiments must be carefully designed and tested. Evaluation of the method for use in regulation will be facilitated if practitioners share their methods and results.

## Footnotes

1 For the purposes of our discussion, species refers to formally identified species as well as to genetically distinct strains or ecotypes (cf. Kashtan et al. 2014).

## References

[CR1] Agustí S, Sánchez MC (2002). Cell viability in natural phytoplankton communities quantified by a membrane permeability probe. Limnol Oceanog.

[CR2] Albert RJ, Lishman JM, Saxena JR (2013). Ballast water regulations and the move toward concentration-based numeric discharge limits. Ecol Appl.

[CR3] Allen E (1919). A contribution to the quantitative study of plankton. J Mar Biol Assoc U K (New Series).

[CR4] Allen E, Nelson E (1910). On the artificial culture of marine plankton organisms. J Mar Biol Assoc U K (New Series).

[CR5] Andersen RA (2005). Algal culturing techniques.

[CR6] Andersen RA, Kawachi M, Andersen RA (2005). Traditional microalgae isolation techniques. Algal culturing techniques.

[CR7] Andersen P, Throndsen J, Hallegraeff GM, Anderson DM, Cembella AD (2003). Estimating cell numbers. Manual on harmful marine microalgae.

[CR8] Backe-Hansen P, Throndsen J (2002). Pico- and nanoplankton from the inner Oslofjord, eastern Norway, including description of two new species of *Luffisphaera* (incerta sedis). Sarsia.

[CR9] Bannister TT, Laws EA, Falkowski PG (1980). Modeling phytoplankton carbon metabolism. Primary Productivity in the Sea.

[CR10] Baroli I, Melis A (1996). Photoinhibition and repair in *Dunaliella salina* acclimated to different growth irradiances. Planta.

[CR11] Behrenfeld MJ, Falkowski PG (1997). A consumer's guide to phytoplankton primary productivity models. Limnol Oceanog.

[CR12] Binder BJ, Anderson DM (1987). Physiological and environmental-control of germination in *Scrippsiella trochoidea* (Dinophyceae) resting cysts. J Phycol.

[CR13] Bird RE, Riordan C (1986). Simple solar spectral model for direct and diffuse irradiance on horizontal and tilted planes at the Earth's surface for cloudless atmospheres. Climate Appl Meteorol.

[CR14] Blanchard GF, Guarini J-M, Richard P, Gros P, Mornet F (1996). Quantifying the short-term temperature effect on light-saturated photosynthesis of intertidal microalgae. Mar Ecol Prog Ser.

[CR15] Blodgett R (2010) Bacteriological Analytical Manual Appendix 2. Most Probable Number from Serial Dilutions. http://www.fda.gov/Food/FoodScienceResearch/LaboratoryMethods/ucm109656.htm. Accessed 26 Nov 2014

[CR16] Boelen P, Veldhuis MJ, Buma AG (2001). Accumulation and removal of UVBR-induced DNA damage in marine tropical plankton subjected to mixed and simulated non-mixed conditions. Aquat Microbial Ecol.

[CR17] Boyce DG, Lewis MR, Worm B (2010). Global phytoplankton decline over the past century. Nature.

[CR18] Brand L, Guillard R (1981). The effects of continuous light and light intensity on the reproduction rates of twenty-two species of marine phytoplankton. J Exp Mar Biol Ecol.

[CR19] Brand LE, Guillard RRL (1981). A method for the rapid and precise determination of acclimated phytoplankton reproduction rates. J Plankton Res.

[CR20] Bravo I, Anderson DM (1994). The effects of temperature, growth-medium and darkness on excystment and growth of the toxic dinoflagellate *Gymnodinium catenatum* from northwest Spain. J Plankton Res.

[CR21] Buma AGJ, Van Hannen EJ, Roza L, Veldhuis MJW, Gieskes WWC (1995). Monitoring ultraviolet-B-induced DNA damage in individual diatom cells by immunofluorescent thymine dimer detection. J Phycol.

[CR22] Burkholder JM, Hallegraeff GM, Melia G, Cohen A, Bowers HA, Oldach DW, Parrow MW, Sullivan MJ, Zimba PV, Allen EH (2007). Phytoplankton and bacterial assemblages in ballast water of US military ships as a function of port of origin, voyage time, and ocean exchange practices. Harmful Algae.

[CR23] Campbell DA, Hossain Z, Cockshutt AM, Zhaxybayeva O, Wu HY, Li G (2013). Photosystem II protein clearance and FtsH function in the diatom *Thalassiosira pseudonana*. Photosynth Res.

[CR24] Cerino F, Zingone A (2006). A survey of cryptomonad diversity and seasonality at a coastal Mediterranean site. Eur J Phycol.

[CR25] Coast Guard US (2012). Standards for living organisms in ships’ ballast water discharged in US waters. Fed Regist.

[CR26] Cochran WG (1950). Estimation of bacterial densities by means of the "most probable number". Biometrics.

[CR27] Cullen JJ (1982). The deep chlorophyll maximum: comparing vertical profiles of chlorophyll a. Can J Fish Aquat Sci.

[CR28] Cullen JJ (1990). On models of growth and photosynthesis in phytoplankton. Deep-Sea Res.

[CR29] Cullen JJ (2001) Plankton: primary production methods. In: Steele J, Thorpe S, Turekian K (eds) Encyclopedia of Ocean Sciences. Academic Press, pp 2277–2284

[CR30] Cullen JJ, MacIntyre JG, Anderson DM, Cembella AD, Hallegraeff GM (1998). Behavior, physiology and the niche of depth-regulating phytoplankton. Physiological Ecology of Harmful Algal Blooms, vol G 41.

[CR31] Cullen JJ, Geider RJ, Ishizaka J, Kiefer DA, Marra J, Sakshaug E, Raven JA, Evans GT, Fasham MJR (1993). Toward a general description of phytoplankton growth for biogeochemical models. Towards a Model of Ocean Biogeochemical Processes.

[CR32] Cullen JJ, Franks PJS, Karl DM, Longhurst A (2002) Physical influences on marine ecosystem dynamics. In: Robinson AR, McCarthy JJ, Rothschild BJ (eds) The Sea: Biological-Physical Interactions in the Ocean, vol 12. Wiley pp 297–335

[CR33] Cullen JJ, Davis RF, Huot Y (2012). Spectral model of depth-integrated water column photosynthesis and its inhibition by ultraviolet radiation. Glob Biogeochem Cycles.

[CR34] Drake LA, Tamburri MN, First MR, Smith GJ, Johengen TH (2014). How many organisms are in ballast water discharge? A framework for validating and selecting compliance monitoring tools. Mar Poll Bull.

[CR35] EPA (2010) Generic protocol for the verification of ballast water treatment technology., Version 5.1. EPA/600/R-10/146. US Environmental Protection Agency Environmental Technology Verification Program, Washington, DC

[CR36] ETV (2010). Generic protocol for the verification of ballast water treatment technology.

[CR37] Fiala M, Oriol L (1990). Light-temperature interactions on the growth of Antarctic diatoms. Polar Biol.

[CR38] First MR, Drake LA (2013). Approaches for determining the effects of UV radiation on microorganisms in ballast water. Manag Biol Inv.

[CR39] First MR, Drake LA (2013). Life after treatment: detecting living microorganisms following exposure to UV light and chlorine dioxide. J Appl Phycol.

[CR40] Follows MJ, Dutkiewicz S (2011). Modeling diverse communities of marine microbes. Annu Rev Mar Sci.

[CR41] Follows MJ, Dutkiewicz S, Grant S, Chisholm SW (2007). Emergent biogeography of microbial communities in a model ocean. Science.

[CR42] Fork DC, Murata N, Sato N (1979). Effect of growth temperature on the lipid and fatty acid composition, and the dependence on temperature of light-induced redox reactions of cytochrome *f* and of light energy redistribution in the thermophilic blue-green alga *Synechococcus lividus*. Plant Physiol.

[CR43] Frazier M, Miller AW, Lee H, Reusser DA (2013). Counting at low concentrations: the statistical challenges of verifying ballast water discharge standards. Ecol Appl.

[CR44] Friedrichs MAM, Carr ME, Barber RT, Scardi M, Antoine D, Armstrong RA, Asanuma I, Behrenfeld MJ, Buitenhuis ET, Chai F, Christian JR, Ciotti AM, Doney SC, Dowell M, Dunne J, Gentili B, Gregg W, Hoepffner N, Ishizaka J, Kameda T, Lima I, Marra J, Melin F, Moore JK, Morel A, O'Malley RT, O'Reilly J, Saba VS, Schmeltz M, Smyth TJ, Tjiputra J, Waters K, Westberry TK, Winguth A (2009). Assessing the uncertainties of model estimates of primary productivity in the tropical Pacific Ocean. J Mar Syst.

[CR45] Furuya K, Marumo R (1983). Size distribution of phytoplankton in the Western Pacific Ocean and adjacent waters in summer. Bull Plankton Soc Japan.

[CR46] Gao Y, Smith GJ, Alberte RS (2000). Temperature dependence of nitrate reductase activity in marine phytoplankton: biochemical analysis and ecological implications. J Phycol.

[CR47] Garthright W, Blodgett R (2003). FDA's preferred MPN methods for standard, large or unusual tests, with a spreadsheet. Food Microbiol.

[CR48] Garvey M, Moriceau B, Passow U (2007). Applicability of the FDA assay to determine the viability of marine phytoplankton under different environmental conditions. Mar Ecol Prog Ser.

[CR49] Geider RJ, MacIntyre HL, Kana TM (1998). A dynamic regulatory model of phytoplankton acclimation to light, nutrient, and temperature. Limnol Oceanog.

[CR50] Gieskes W, Kraay G (1983). Dominance of Cryptophyceae during the phytoplankton spring bloom in the central North Sea detected by HPLC analysis of pigments. Mar Biol.

[CR51] Giovannoni SJ, Britschgi TB, Moyer CL, Field KG (1990). Genetic diversity in Sargasso Sea bacterioplankton. Nature.

[CR52] Granéli E, Salomon PS (2010). Factors influencing allelopathy and toxicity in *Prymnesium parvum*. J Am Water Resour Assoc.

[CR53] Gregg MD, Hallegraeff GM (2007). Efficacy of three commercially available ballast water biocides against vegetative microalgae, dinoflagellate cysts and bacteria. Harmful Algae.

[CR54] Guillard R, Andersen RA (2005). Purification methods for microalgae. Algal culturing techniques.

[CR55] Guillard R, Morton S (2003) Culture methods. Manual on harmful marine microalgae. UNESCO IOC Monographs on Oceanographic Methodology 11:77–97

[CR56] Haas CN, Heller B (1988). Test of the validity of the Poisson assumption for analysis of most-probable-number results. Appl Environ Microbiol.

[CR57] Hancke K, Hancke TB, Olsen LM, Johnsen G, Glud RN (2008). Temperature effects on microalgal photosynthesis-light responses measured by O_2_ production, pulse-amplitude-modulated fluorescence, and ^14^C assimilation. J Phycol.

[CR58] Hardison DR, Sunda WG, Shea D, Litaker RW (2013) Increased toxicity of Karenia brevis during phosphate limited growth: ecological and evolutionary implications. Plos One 8(3). doi:10.1371/journal.pone.0058545 10.1371/journal.pone.0058545 PMC3595287 23554901

[CR59] Harrison WG, Platt T (1980). Variations in assimilation number of coastal marine phytoplankton: effects of environmental covariates. J Plankton Res.

[CR60] Harrison WG, Platt T (1986). Photosynthesis–irradiance relationships in polar and temperate phytoplankton populations. Polar Biol.

[CR61] Heywood JL, Sieracki ME, Bellows W, Poulton NJ, Stepanauskas R (2010). Capturing diversity of marine heterotrophic protists: one cell at a time. ISME J.

[CR62] Hijnen W, Beerendonk E, Medema GJ (2006). Inactivation credit of UV radiation for viruses, bacteria and protozoan (oo)cysts in water: a review. Water Res.

[CR63] Hikosaka K, Ishikawa K, Borjigidai A, Muller O, Onoda Y (2006). Temperature acclimation of photosynthesis: mechanisms involved in the changes in temperature dependence of photosynthetic rate. J Exp Bot.

[CR64] Hulburt EM (1982). The adaptation of marine phytoplankton species to nutrient and temperature. Ocean Sci Eng.

[CR65] Humphries S, Widjaja F (1979). A simple method for separating cells of *Microcystis aeruginosa* for counting. Brit Phycol J.

[CR66] Hurley MA, Roscoe M (1983). Automated statistical analysis of microbial enumeration by dilution series. J Appl Bact.

[CR67] IMO (2004) Convention BWM/CONF/36 International Convention for the Control and Management of Ships’ Ballast Water and Sediments

[CR68] IMO (2015). http://www.imo.org/OurWork/Environment/BallastWaterManagement/Documents/Table of BA FA TA updated in Oct 2014.pdf Accessed 14 Jan 2015

[CR69] International Maritime Organization Marine Environment Protection Committee (2008) Annex 4 Resolution MEPC. 174 (58). Guidelines for Approval of Ballast Water Management Systems (G8). London, UK

[CR70] Ishikawa A, Furuya K (2004). The role of diatom resting stages in the onset of the spring bloom in the East China Sea. Mar Biol.

[CR71] Jochem FJ (1990). On the seasonal occurrence of autotrophic naked nanoflagellates in Kiel Bight, western Baltic. Estuar Coast Shelf Sci.

[CR72] Johnson ZI, Zinser ER, Coe A, McNulty NP, Woodward EMS, Chisholm SW (2006). Niche partitioning among *Prochlorococcus* ecotypes along ocean-scale environmental gradients. Science.

[CR73] Kaeberlein T, Lewis K, Epstein SS (2002). Isolating "uncultivable" microorganisms in pure culture in a simulated natural environment. Science.

[CR74] Kashtan N, Roggensack SE, Rodrigue S, Thompson JW, Biller SJ, Coe A, Ding H, Marttinen P, Malmstrom RR, Stocker R, Follows MJ, Stepanauskas R, Chisholm SW (2014). Single-cell genomics reveals hundreds of coexisting subpopulations in wild *Prochlorococcus*. Science.

[CR75] Kawachi M, Noël M-H, Andersen RA (2005). Sterilization and sterile technique. Algal culturing techniques.

[CR76] King DM, Tamburri MN (2010). Verifying compliance with ballast water discharge regulations. Ocean Dev Int Law.

[CR77] Knight-Jones E (1951). Preliminary studies of nanoplankton and ultraplankton systematics and abundance by a quantitative culture method. J Conseil.

[CR78] Krupke A, Lavik G, Halm H, Fuchs BM, Amann RI, Kuypers MMM (2014). Distribution of a consortium between unicellular algae and the N_2_ fixing cyanobacterium UCYN-A in the North Atlantic Ocean. Env Microbiol.

[CR79] Kugrens P, Lee RE (1987). An ultrastructural survey of cryptomonad periplasts using quick‐freezing freeze fracture techniques. J Phycol.

[CR80] Landry MR, Hassett RP (1982). Estimating the grazing impact of marine micro-zooplankton. Mar Biol.

[CR81] Letelier RM, Karl DM, Abbott MR, Bidigare RR (2004). Light driven seasonal patterns of chlorophyll and nitrate in the lower euphotic zone of the North Pacific Subtropical Gyre. Limnol Oceanogr.

[CR82] Li WKW, Dickie PM (2001). Monitoring phytoplankton, bacterioplankton, and virioplankton in a coastal inlet (Bedford Basin) by flow cytometry. Cytometry.

[CR83] Liebich V, Stehouwer PP, Veldhuis M (2012). Re-growth of potential invasive phytoplankton following UV-based ballast water treatment. Aquat Invas.

[CR84] Lighthart B (1969). Planktonic and benthic bacteriovorous protozoa at eleven stations in Puget Sound and adjacent Pacific Ocean. J Fish Board Canada.

[CR85] Lima ID, Doney SC (2004). A three-dimensional, multi-nutrient, and size-structured ecosystem model for the North Atlantic. Glob Biogeochem Cycles.

[CR86] MacIntyre HL, Kana TM, Anning T, Geider RJ (2002). Photoacclimation of photosynthesis irradiance response curves and photosynthetic pigments in microalgae and cyanobacteria. J Phycol.

[CR87] Marañón E (2015). Cell size as a key determinant of phytoplankton metabolism and community structure. Annu Rev Mar Sci.

[CR88] McCrady MH (1915). The numerical interpretation of fermentation-tube results. J Infect Dis.

[CR89] Montagnes DJ, Berges JA, Harrison PJ, Taylor F (1994). Estimating carbon, nitrogen, protein, and chlorophyll a from volume in marine phytoplankton. Limnol Oceanog.

[CR90] Moore LR, Post AF, Rocap G, Chisholm SW (2002). Utilization of different nitrogen sources by the marine cyanobacteria *Prochlorococcus* and *Synechococcus*. Limnol Oceanog.

[CR91] Moore LR, Coe A, Zinser ER, Saito MA, Sullivan MB, Lindell D, Frois-Moniz K, Waterbury J, Chisholm SW (2007). Culturing the marine cyanobacterium *Prochlorococcus*. Limnol Oceanogr Methods.

[CR92] Neale PJ, Banaszak AT, Jarriel CR (1998). Ultraviolet sunscreens in *Gymnodinium sanguineum* (Dinophyceae): mycosporine‐like amino acids protect against inhibition of photosynthesis. J Phycol.

[CR93] Nielsen LT, Krock B, Hansen PJ (2013). Production and excretion of okadaic acid, pectenotoxin-2 and a novel dinophysistoxin from the DSP-causing marine dinoflagellate *Dinophysis acuta*—effects of light, food availability and growth phase. Harmful Algae.

[CR94] Nitta K, Suzuki N, Honman D, Kaneko Y, Nakamoto H (2006). Ultrastructural stability under high temperature or intensive light stress conferred by a small heat shock protein in cyanobacteria. FEBS Lett.

[CR95] Norberg J (2004). Biodiversity and ecosystem functioning: a complex adaptive systems approach. Limnol Oceanog.

[CR96] Oemcke D, Van Leeuwen J (2005). Ozonation of the marine dinoflagellate alga *Amphidinium* sp.—implications for ballast water disinfection. Water Res.

[CR97] Paasche E (1968). Marine plankton algae grown with light–dark cycles. II. *Ditylum brightwellii* and *Nitzschia turgidula*. Physiol Plant.

[CR98] Paerl HW (1978). Effectiveness of various counting methods in detecting viable phytoplankton. N Z J Mar Freshw Res.

[CR99] Park MG, Kim S, Kim HS, Myung G, Kang YG, Yih W (2006). First successful culture of the marine dinoflagellate *Dinophysis acuminata*. Aquat Microbial Ecol.

[CR100] Partensky F, Hess W, Vaulot D (1999). *Prochlorococcus*, a marine photosynthetic prokaryote of global significance. Microbiol Molec Biol Rev.

[CR101] Peperzak L, Brussaard CPD (2011). Flow cytometric applicability of fluorescent vitality probes on phytoplankton. J Phycol.

[CR102] Platt T, Sathyendranath S (1993). Estimators of primary production for the interpretation of remotely-sensed data on ocean color. J Geophys Res.

[CR103] Rappé MS, Connon SA, Vergin KL, Giovannoni SJ (2002). Cultivation of the ubiquitous SAR11 marine bacterioplankton clade. Nature.

[CR104] Reavie ED, Cangelosi AA, Allinger LE (2010). Assessing ballast water treatments: Evaluation of viability methods for ambient freshwater microplankton assemblages. J Great Lakes Res.

[CR105] Revelante N, Gilmartin M (1991). The phytoplankton composition and population enrichment in gelatinous macroaggregates in the northern Adriatic during the summer of 1989. J Exp Mar Biol Ecol.

[CR106] Richardson K (1991). Comparison of ^14^C primary production determinations made by different laboratories. Mar Ecol Prog Ser.

[CR107] Ritchie RJ, Larkum AWD (2012). Modelling photosynthesis in shallow algal production ponds. Photosynthetica.

[CR108] Rodriguez-Martinez R, Rocap G, Salazar G, Massana R (2013). Biogeography of the uncultured marine picoeukaryote MAST-4: temperature-driven distribution patterns. Isme J.

[CR109] Roy S, de Mora S, Demers S, Vernet M (2000). Strategies for the minimisation of UV-induced damage. The Effects of UV Radiation in the Marine Environment.

[CR110] Saba VS, Friedrichs MAM, Carr M-E, Antoine D, Armstrong RA, Asanuma I, Aumont O, Bates NR, Behrenfeld MJ, Bennington V, Bopp L, Bruggeman J, Buitenhuis ET, Church MJ, Ciotti AM, Doney SC, Dowell M, Dunne J, Dutkiewicz S, Gregg W, Hoepffner N, Hyde KJW, Ishizaka J, Kameda T, Karl DM, Lima I, Lomas MW, Marra J, McKinley GA, Mélin F, Moore JK, Morel A, O'Reilly J, Salihoglu B, Scardi M, Smyth TJ, Tang S, Tjiputra J, Uitz J, Vichi M, Waters K, Westberry TK, Yool A (2010). Challenges of modeling depth-integrated marine primary productivity over multiple decades: a case study at BATS and HOT. Global Biogeochem Cycl.

[CR111] Selvin R, Requera B, Bravo I, Yentsch CM (1988). Use of fluorescein diacetate (FDA) as a single-cell probe of metabolic activity in dinoflagellate cultures. Biol Oceanogr.

[CR112] Sherr EB, Sherr BF (2007). Heterotrophic dinoflagellates: a significant component of microzooplankton biomass and major grazers of diatoms in the sea. Mar Ecol Prog Ser.

[CR113] Shi XL, Marie D, Jardillier L, Scanlan DJ, Vaulot D (2009) Groups without cultured representatives dominate eukaryotic picophytoplankton in the oligotrophic South East Pacific Ocean. Plos One 4(10). doi:10.1371/journal.pone.0007657 10.1371/journal.pone.0007657 PMC2764088 19893617

[CR114] Sieracki M, Poulton N, Crosbie N, Andersen RA (2005). Automated isolation techniques for microalgae. Algal culturing techniques.

[CR115] Sinigalliano CD, Winshell J, Guerrero MA, Scorzetti G, Fell JW, Eaton RW, Brand L, Rein KS (2009). Viable cell sorting of dinoflagellates by multiparametric flow cytometry. Phycologia.

[CR116] Smayda TJ (1969). Experimental observations on the influence of temperature, light, and salinity on cell division of the marine diatom *Detonula confervacea* (Cleve) Gran. J Phycol.

[CR117] Steinberg MK, Lemieux EJ, Drake LA (2011). Determining the viability of marine protists using a combination of vital, fluorescent stains. Mar Biol.

[CR118] Steinberg MK, First MR, Lemieux EJ, Drake LA, Nelson BN, Kulis DM, Anderson DM, Welschmeyer NA, Herring PR (2012). Comparison of techniques used to count single-celled viable phytoplankton. J Appl Phycol.

[CR119] Strom SL, Miller CB, Frost BW (2000). What sets lower limits to phytoplankton stocks in high-nitrate, low-chlorophyll regions of the open ocean?. Mar Ecol Prog Ser.

[CR120] Sundström AM, Kremp A, Daugbjerg N, Moestrup O, Ellegaard M, Hansen R, Hajdu S (2009). *Gymnodinium corollarium sp nov* (Dinophyceae)—a new cold-water dinoflagellate responsible for cyst sedimentation events in the Baltic Sea. J Phycol.

[CR121] Suttle CA, Chan AM (1993). Marine cyanophages infecting oceanic and coastal strains of Synechococcus: abundance, morphology, cross-infectivity and growth characteristics. Mar Ecol Prog Ser.

[CR122] Talling JF (1957). The phytoplankton population as a compound photosynthetic system. New Phytol.

[CR123] Tameishi M, Yamasaki Y, Nagasoe S, Shimasaki Y, Oshima Y, Honjo T (2009). Allelopathic effects of the dinophyte Prorocentrum minimum on the growth of the bacillariophyte *Skeletonema costatum*. Harmful Algae.

[CR124] Thomas WH (1966). Effects of temperature and illuminance on cell division rates of three species of tropical oceanic phytoplankton. J Phycol.

[CR125] Thomas MK, Kremer CT, Klausmeier CA, Litchman E (2012). A global pattern of thermal adaptation in marine phytoplankton. Science.

[CR126] Throndsen J, Sournia A (1978). The dilution-culture method. Phytoplankton manual, vol 6.

[CR127] Throndsen J, Kristiansen S (1991). *Micromonas pusilla* (Prasinophyceae) as part of pico‐ and nanoplankton communities of the Barents Sea. Polar Res.

[CR128] Tyystjärvi E (2008). Photoinhibition of Photosystem II and photodamage of the oxygen evolving manganese cluster. Coord Chem Rev.

[CR129] U.S. Environmental Protection Agency Environmental Technology Verification Program (2010) Generic protocol for the verification of ballast water treatment technology. Report number EPA/600/R–10/146

[CR130] Usup G, Kulis DM, Anderson DM (1994). Growth and toxin production of the toxic dinoflagellate *Pyrodinium bahamense var. compressum* in laboratory cultures. Nat Toxins.

[CR131] van Reis R, Brake JM, Charkoudian J, Burns DB, Zydney AL (1999). High-performance tangential flow filtration using charged membranes. J Membr Sci.

[CR132] Vaulot D, Eikrem W, Viprey M, Moreau H (2008). The diversity of small eukaryotic phytoplankton (≤3 μm) in marine ecosystems. FEMS Microbiol Rev.

[CR133] Verity PG, Smetacek V (1996). Organism life cycles, predation, and the structure of marine pelagic ecosystems. Mar Ecol Prog Ser.

[CR134] Wood AM, Everroad RC, Wingard LM, Andersen RA (2004). Measuring growth rates in algal cultures. Algal culturing techniques.

[CR135] Wright D, Dawson R, Caceres V, Orano‐Dawson C, Kananen G, Cutler S, Cutler H (2009). Shipboard testing of the efficacy of SeaKleen® as a ballast water treatment to eliminate non‐indigenous species aboard a working tanker in Pacific waters. Env Technol.

[CR136] Yoder JA (1979). Effect of temperature on light-limited growth and chemical composition of *Skeletonema costatum* (Bacillariophyceae). J Phycol.

[CR137] Zehr JP, Waterbury JB, Turner PJ, Montoya JP, Omoregie E, Steward GF, Hansen A, Karl DM (2001). Unicellular cyanobacteria fix N_2_ in the subtropical North Pacific Ocean. Nature.

[CR138] Zetsche E-M, Meysman FJR (2012). Dead or alive? Viability assessment of micro- and mesoplankton. J Plankton Res.

